# Natural product-derived interventions for reversing multidrug resistance in resistant K562 leukemia cells: a systematic review and meta-analysis

**DOI:** 10.3389/fphar.2026.1892591

**Published:** 2026-09-25

**Authors:** Jingyi Sun, Changning Song, Yining Hua, Jiayin Pang, Shuaiting Sun

**Affiliations:** School of Biomedical and Chemical Engineering, Liaoning Institute of Science and Technology, Benxi, China

**Keywords:** K562 leukemia, meta-analysis, multidrug resistance, natural products, reversal fold

## Abstract

**Background:**

Multidrug resistance remains a major barrier to effective chemotherapy for leukemia. Natural product-derived interventions have been widely investigated as modulators of drug resistance in adriamycin- or doxorubicin-resistant K562 cell models, but their quantitative reversal effects remain heterogeneous.

**Methods:**

*In vitro* studies evaluating natural product-derived interventions in resistant K562 leukemia sublines were synthesized. The primary meta-analytic endpoint was IC50-based reversal fold (RF), analyzed as log (RF) using an inverse-variance random-effects model. Subgroup, sensitivity, leave-one-out, and small-study-effect analyses were performed. Mechanistic outcomes related to transporter modulation, drug accumulation, apoptosis, and signaling pathways were summarized descriptively.

**Results:**

Twenty-eight *in vitro* studies were included, and 19 IC50-based study-level effects were entered into the primary meta-analysis. The pooled RF was 6.31 (95% CI: 4.77–8.36), representing an overall chemosensitization signal across the restricted quantitative dataset. Subgroup analysis showed pooled RF values of 5.71 for K562/A02, 4.90 for K562/ADM, and 8.91 for K562/ADR. Heterogeneity was high (I^2^ = 97.7%), indicating substantial variability across resistant-cell sublines, intervention types, substrate drugs, and experimental conditions. Sensitivity analyses confirmed that the pooled effect remained positive, although exclusion of high-effect studies reduced the pooled RF to 4.51. Trim-and-fill analysis produced an adjusted pooled RF of 5.48 (95% CI: 3.87–7.76). Across individual studies, reported mechanisms included ABC transporter modulation, increased intracellular anthracycline accumulation, reduced efflux, restoration of apoptosis, and modulation of PI3K/Akt, NF-κB, and Nrf2/ARE signaling.

**Conclusion:**

Across the heterogeneous studies, the common IC50-based endpoint showed an overall chemosensitization signal; however, this pooled summary does not imply a shared pharmacological action or potency across chemically distinct metabolites. Effect magnitude and mechanisms remained specific to individual metabolites, resistant-cell sublines, substrate drugs, doses, and molecular targets.

## 1 Introduction

Multidrug resistance (MDR) remains a major experimental and therapeutic barrier in leukemia chemotherapy, particularly in resistant K562-derived models used to study acquired resistance to adriamycin and doxorubicin. In these models, resistant sublines such as K562/A02, K562/ADM, and K562/ADR are widely used to evaluate whether candidate agents can restore intracellular drug retention, reduce IC50 values, and resensitize cells to anthracycline-based cytotoxicity (Li et al., 2012; Zhang et al., 2013; Chen et al., 2016). Because these sublines reproduce key features of drug-resistant leukemia cells, they provide a useful *in vitro* platform for investigating both transporter-mediated efflux and broader resistance-associated signaling mechanisms. The continuous search for novel chemotherapeutic agents has led to systematic reviews of various chemical derivatives, such as s-triazine derivatives, for their antitumor activities (Dai et al., 2023).

K562 cells are derived from chronic myeloid leukemia and therefore do not fully recapitulate current clinical management of CML, which is dominated by BCR-ABL1 tyrosine kinase inhibitors such as imatinib. Acquired resistance to targeted therapies is also a major challenge in other malignancies, such as non-small cell lung cancer resistance to EGFR-TKIs mediated by non-coding RNAs (Wen et al., 2023; Zheng et al., 2025). However, the rationale for using resistant K562 sublines in this review is specifically experimental rather than therapeutic-first-line modeling. K562/ADM, K562/ADR, and K562/A02 cells are established *in vitro* systems for studying acquired multidrug resistance, particularly ABC transporter activity, anthracycline efflux, intracellular drug retention, apoptosis escape, and resistance-associated survival signaling. Therefore, these models are appropriate for evaluating whether natural product-derived interventions can modulate MDR mechanisms. Still, the findings should not be interpreted as direct evidence to replace or compete with TKI-based CML therapy.

The most frequently investigated mechanism in adriamycin- or doxorubicin-resistant K562 cells is ATP-binding cassette transporter-mediated drug efflux, especially P-gp/ABCB1 (MDR1). Earlier K562/A02 and K562/ADR studies consistently linked resistance reversal to reduced P-gp activity, increased ADR/DOX or Rh123 accumulation, or suppression of MDR1/P-gp expression (Li et al., 2012; Zhang et al., 2013; Ji et al., 2011; Shu-Qi et al., 2011; Wang et al., 2011; Wang S.-Q. et al., 2016). However, MDR in these models is not exclusively P-gp-dependent. MRP1/ABCC1 has been implicated in wogonin- and timosaponin A-III-mediated reversal, whereas BCRP/ABCG2 and additional resistance-associated transporters have been reported in later studies involving more complex resistant phenotypes (Chen et al., 2016; Xu et al., 2014; Wang et al., 2022; Yi et al., 2021; Shen et al., 2025; Wang et al., 2025a). Therefore, MDR reversal in K562 cells should be understood as a transporter-centered, yet mechanistically heterogeneous, phenomenon.

Natural product-derived interventions have attracted sustained interest as MDR modulators because of their chemical diversity and capacity to act through multiple biological mechanisms. The structural diversity of natural products requires advanced analytical approaches for dereplication and identification, such as integrated molecular networking workflows (Sheng et al., 2024). The eligible studies in this review investigated structurally diverse agents, including marine or shellfish-derived protein extract, diterpenoids, bisbibenzyl phenolics, coumarins, flavonoids, steroidal saponins, anthraquinones, glycoalkaloids, quassinoids, lignans, sesquiterpene lactones, triterpenoids, diarylheptanoids, and fungal metabolites. Several of these metabolites were evaluated as alternatives or comparators to classical MDR-modulating agents such as verapamil, which have been limited by toxicity, pharmacokinetic constraints, or poor translational suitability in prior MDR-reversal contexts (Li et al., 2012; Zhang et al., 2013; Ji et al., 2011; Shu-Qi et al., 2011; Wang et al., 2011). This pharmacological diversity supports summarizing a common assay-level chemosensitization endpoint while retaining metabolite-specific pharmacological interpretation rather than treating MDR reversal as a uniform class effect.

Mechanistically, natural product-mediated MDR reversal in resistant K562 models extends beyond direct efflux inhibition. Some agents primarily inhibit transporter function, as shown by increased intracellular ADR/DOX or Rh123 accumulation after treatment with riccardin F, pakyonol, and Euphorbia factor L1 (Zhang et al., 2013; Ji et al., 2011; Wang W.-z. et al., 2024). Other interventions reduce transporter expression or transcriptional activity, including archangelicin-mediated MDR1/P-gp downregulation, wogonin-mediated MRP1 suppression through Nrf2/ARE inhibition, and timosaponin A-III-mediated reduction of MDR1 and MRP1 through PI3K/Akt pathway inhibition (Chen et al., 2016; Shu-Qi et al., 2011; Xu et al., 2014). Additional studies link MDR reversal to apoptosis restoration, autophagy-associated mechanisms, NF-κB/IκB-α signaling, PI3K/Akt/mTOR signaling, JNK-associated regulation, CXCL12/CXCR4 survival signaling, and Nrf2-centered resistance control (Li et al., 2012; Zhang et al., 2013; Chen et al., 2016; Xu et al., 2014; Wang et al., 2022; Yi et al., 2021; Shen et al., 2025; Li et al., 2010; Wang Y. et al., 2014; Min et al., 2017; Cai et al., 2019; Liu et al., 2021; Wang L. et al., 2016). These overlapping mechanisms necessitate a structured synthesis.

An ethnopharmacological rationale also underpins the present synthesis, as many of the reviewed MDR-modulating metabolites are derived from medicinal plants, fungi, or other natural sources with documented traditional use. Representative validated sources include *Ailanthus altissima* (Mill.) Swingle [Simaroubaceae], *Scutellaria baicalensis* Georgi [Lamiaceae], *Pueraria montana* var. *lobata* (Willd.) Maesen and S.M.Almeida ex Sanjappa & Predeep [Fabaceae], *Cnidium monnieri* (L.) Cusson [Apiaceae], *Rheum palmatum* L. [Polygonaceae], *Anemarrhena asphodeloides* Bunge [Asparagaceae], *Schisandra chinensis* (Turcz.) Baill. [Schisandraceae], *Ganoderma lucidum* (Curtis) P.Karst. [Ganodermataceae], and *Tripterygium wilfordii* Hook.f. [Celastraceae]; for *Dioscorea*, the original evidence did not consistently permit species-level assignment, so the source is retained as *Dioscorea* spp. [Dioscoreaceae] without inference. These sources have been described in traditional or ethnomedicinal systems for inflammatory, chronic, metabolic, febrile, tonic, or disease-resistance-related indications (Li et al., 2021; Zhao et al., 2019; Miao et al., 2025; Lin et al., 2020). Although these traditional uses do not directly correspond to multidrug resistance or K562 leukemia models, they provide a source-based pharmacological rationale for examining whether their bioactive metabolites can modulate experimentally defined resistance mechanisms, including ABC-transporter activity, intracellular anthracycline retention, apoptosis restoration, autophagy regulation, and NF-κB, PI3K/Akt, and Nrf2/ARE signaling pathways.

Despite the number of individual *in vitro* studies, the evidence base remains fragmented across different resistant K562 sublines, natural product-derived metabolite classes, dose ranges, exposure durations, assay platforms, and endpoint definitions. Some studies report directly extractable ADM/DOX IC50 or reversal-fold outcomes. In contrast, others provide graph-based sensitization data, non-anthracycline substrate assays, transporter-function outcomes, apoptosis endpoints, or pathway-level evidence. This heterogeneity has limited direct comparisons across studies and left uncertainty about the overall magnitude of natural product-mediated MDR reversal in adriamycin-resistant K562 models.

Therefore, this meta-analysis aimed to synthesize original full-text *in vitro* studies evaluating natural product-derived interventions for reversing MDR in adriamycin- or doxorubicin-resistant K562 cells. The primary objective was to summarize the direction and magnitude of the common ADM/DOX IC50-based reversal endpoint among studies meeting strict comparability criteria; the pooled estimate was not intended to represent a shared mechanism, potency, or class-wide pharmacological effect across chemically distinct metabolites. Secondary objectives were to characterize resistant K562 models, classify natural product-derived interventions, summarize modulation of transporters and resistance markers, evaluate cell death and signaling pathways associated with MDR reversal, and assess methodological concerns influencing quantitative synthesis.

## 2 Methods

### 2.1 Study design and reporting framework

This study was designed as a systematic review and meta-analysis of *in vitro* experimental studies evaluating natural product–derived interventions for reversing multidrug resistance in adriamycin- or doxorubicin-resistant K562 leukemia cells. The methodology followed the Preferred Reporting Items for Systematic Reviews and Meta-Analyses (PRISMA) 2020 framework, with adaptations appropriate for preclinical *in vitro* studies. The review question was structured around four core elements: a resistant K562 cell model, a natural product–derived intervention, an appropriate resistant-cell comparator, and quantitative multidrug resistance-reversal outcomes. The review framework was aligned with the Frontiers Four Pillars of Best Practice for Ethnopharmacology by extracting traditional/source context, pharmacological model, dose and control information, preparation/source information when reported, taxonomic validation, evidence hierarchy, and quality-assessment variables, while restricting interpretation to the eligible *in vitro* K562 MDR evidence.

For the quantitative synthesis, the primary meta-analytic effect size was defined as the log-transformed reversal fold [log (RF)] derived from IC50-based adriamycin/doxorubicin resistance-reversal outcomes, allowing pooled estimation of MDR-reversal effects across eligible studies.

The meta-analysis was considered appropriate only for the restricted subset of studies that reported comparable IC50-based ADM/ADR/DOX reversal-fold outcomes with estimable variance. Because the included natural product-derived interventions differed in chemical class, source material, resistant K562 subline, substrate drug, dose, exposure duration, and dominant mechanism, the pooled estimate was interpreted only as an exploratory summary of a common assay-level MDR-sensitization endpoint, not as evidence for a shared mechanism, potency ranking, or uniform pharmacological effect across metabolites. Studies that were mechanistically informative but insufficiently comparable for RF-based pooling were therefore retained only for structured qualitative synthesis.

### 2.2 Literature search strategy

A comprehensive literature search was conducted to identify eligible full-text original studies published between 1 January 2010 and 31 December 2025. The search covered PubMed/MEDLINE, Scopus, Web of Science Core Collection, Embase, Cochrane Library, ScienceDirect, SpringerLink, Wiley Online Library, Taylor & Francis Online, SAGE Journals, Google Scholar, Dimensions, OpenAlex, CNKI, Wanfang Data, and VIP Chinese Science and Technology Journal Database. Reference lists of eligible full-text articles were also manually screened to identify additional relevant studies.

The search strategy combined terms related to K562 adriamycin/doxorubicin-resistant leukemia cells, multidrug resistance, natural product–derived interventions, and quantitative MDR-reversal outcomes suitable for meta-analysis. The main search terms included: “K562,” “K-562,” “K562/ADM,” “K562/ADR,” “K562/A02,” “adriamycin-resistant K562,” “doxorubicin-resistant K562,” “adriamycin,” “doxorubicin,” “multidrug resistance,” “MDR,” “drug resistance,” “chemoresistance,” “natural product,” “natural compound,” “phytochemical,” “plant extract,” “botanical extract,” “traditional medicine,” “traditional Chinese medicine,” “flavonoid,” “alkaloid,” “polyphenol,” “saponin,” “terpenoid,” “P-glycoprotein,” “P-gp,” “MDR1,” “ABCB1,” “MRP1,” “ABCC1,” “BCRP,” “ABCG2,” “drug efflux,” “intracellular accumulation,” “IC50,” “reversal,” “reversal fold,” “reversal index,” “sensitization,” “chemosensitization,” “standard deviation,” “standard error,” and “confidence interval.” Boolean operators were adapted to each database’s syntax. Review articles, systematic reviews, meta-analyses, editorials, letters, commentaries, conference abstracts, book chapters, theses, dissertations, patents, protocols, and other non-original publications were excluded at both database filtering and manual screening stages.

### 2.3 Eligibility criteria

Studies were included if they met all predefined criteria: original full-text peer-reviewed *in vitro* experimental article; publication between 1 January 2010 and 31 December 2025; use of adriamycin- or doxorubicin-resistant K562 leukemia cells, including K562/ADM, K562/ADR, K562/A02, or clearly defined equivalent resistant sublines; evaluation of a clearly natural product–derived intervention; assessment of multidrug resistance reversal, resistance attenuation, or chemosensitization; inclusion of an appropriate resistant-cell comparator; and reporting of quantitative data relevant to MDR reversal. Studies were eligible for the quantitative meta-analysis when they reported directly extractable or derivable IC50-based reversal-fold data for ADM, ADR, or DOX, together with sufficient information to estimate the log-transformed reversal fold [log (RF)] and its sampling variance, including standard deviation, standard error, confidence interval, number of independent experiments, or graphically extractable error estimates. Studies that met the broader eligibility criteria but lacked sufficient variance information for meta-analysis were retained only for qualitative and mechanistic synthesis. The complete eligibility framework is summarized in Table 1.

**TABLE 1:** Summary of eligibility criteria.

Domain	Inclusion criteria	Exclusion criteria
Publication period	1 January 2010 to 31 December 2025	Studies outside the predefined period
Article type	Full-text peer-reviewed original article	Reviews, systematic reviews, meta-analyses, editorials, letters, conference abstracts, theses, patents, protocols, book chapters
Study model	*In vitro* experimental study	Animal-only, clinical-only, ex vivo-only, computational-only studies
Cell line	K562/ADM, K562/ADR, K562/A02, or clearly defined adriamycin/doxorubicin-resistant K562 cells	Parental K562 only; non-K562 resistant cells without separate K562-resistant data
Intervention	Clearly natural product–derived metabolite, extract, or fraction	Synthetic drugs, gene-editing methods, siRNA/shRNA, antibodies, or pharmaceutical inhibitors as primary intervention
Comparator	Resistant-cell control, vehicle control, ADM/DOX/ADR-only group, or equivalent comparator	No valid comparator group
Qualitative outcome	Quantitative MDR-reversal, resistance-attenuation, chemosensitization, transporter, accumulation, apoptosis, or pathway-related outcome	No MDR-reversal endpoint or insufficient quantitative/mechanistic data
Meta-analysis outcome	Directly extractable or derivable ADM/ADR/DOX IC50-based RF data with sufficient information to estimate log (RF) and its variance	No extractable IC50-based RF, no estimable variance, graph-only data without readable error estimates, or outcomes not comparable with the primary RF endpoint

Studies were excluded if they were published outside the predefined period; were reviews, meta-analyses, editorials, letters, conference abstracts, theses, patents, protocols, or other non-original publications; were not full-text articles; did not use adriamycin- or doxorubicin-resistant K562 cells; used parental K562 cells only; used other resistant cancer cell lines without separately extractable K562-resistant data; did not evaluate MDR reversal; used synthetic, gene-based, antibody-based, or pharmaceutical interventions as the primary MDR-reversing agent; lacked an appropriate comparator; provided insufficient quantitative data for either qualitative synthesis or meta-analysis; reported only molecular docking, network pharmacology, or bioinformatics prediction without *in vitro* validation; contained duplicate or overlapping datasets; or had been retracted. Natural product–nanocarrier formulations were not included in the strict main analysis unless the independent effect of the natural product could be clearly isolated.

### 2.4 Study selection

All retrieved records were imported into a reference management file, and duplicate records were removed prior to screening. Titles and abstracts were first screened against the eligibility criteria. Potentially relevant records were then assessed by full-text review. Studies were selected only when the resistant K562 cell model, natural product–derived intervention, comparator, and extractable MDR-reversal outcome were clearly identifiable. Disagreements during screening were resolved by rechecking the full text against the predefined criteria. The PRISMA-based study-selection flow diagram is shown in Figure 1. In the flow diagram, quantitative synthesis refers to all included studies contributing quantitative experimental or mechanistic MDR-reversal data. In contrast, the primary meta-analysis was restricted to studies with directly extractable or derivable IC50-based reversal-fold data suitable for log (RF)-based pooling.

**FIGURE 1:**
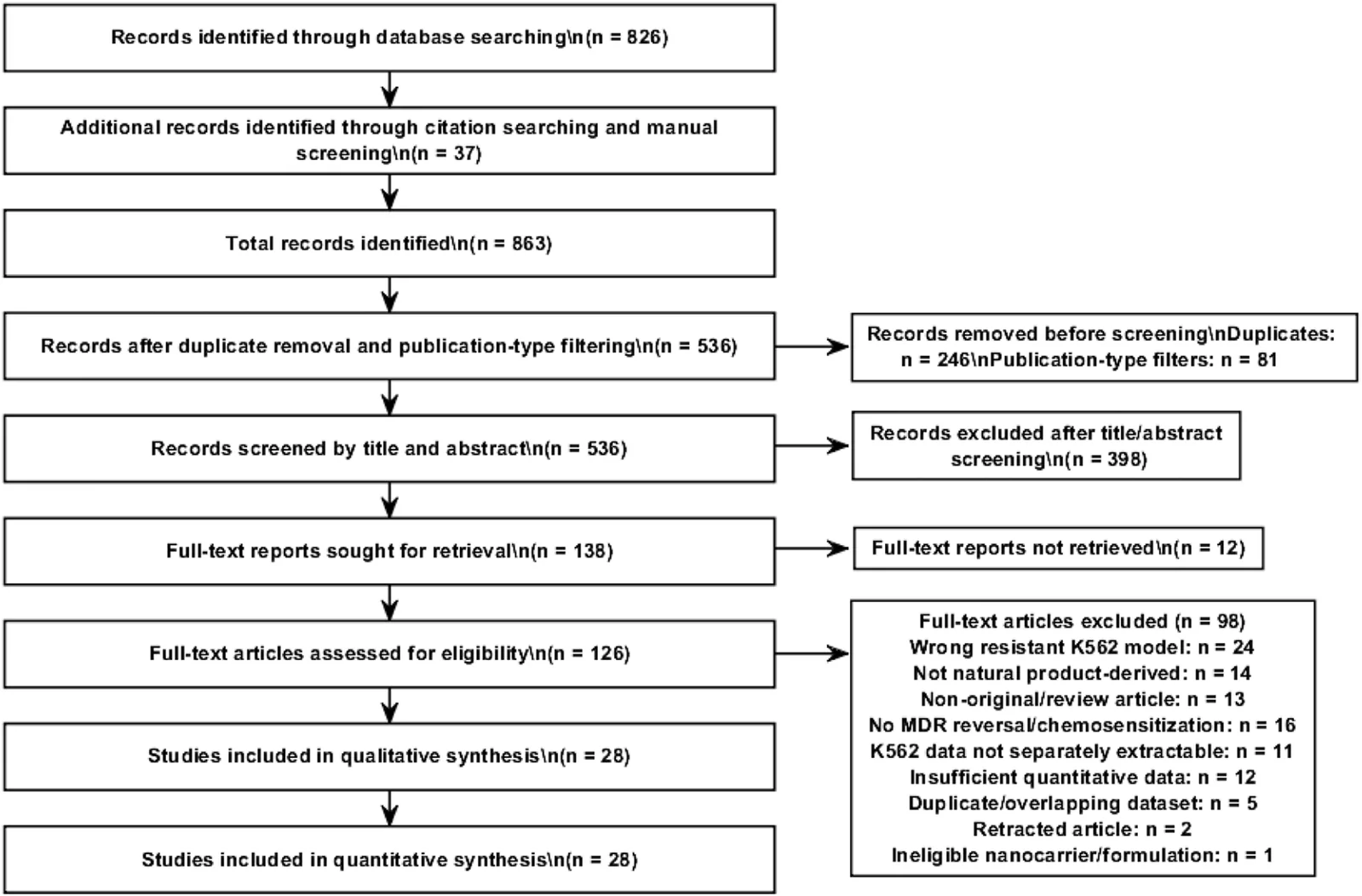
PRISMA-based study-selection flow diagram.

The PRISMA selection process identified 863 records, including 826 from database searching and 37 from citation tracking and manual reference screening. After removing 246 duplicates and excluding 81 records based on publication-type filters, 536 records underwent title and abstract screening. Of these, 398 were excluded as irrelevant to the review question. Full texts were sought for 138 reports, of which 12 could not be retrieved. A total of 126 full-text articles were assessed for eligibility, and 98 were excluded for predefined reasons. Finally, 28 original full-text *in vitro* studies were included in the qualitative and quantitative synthesis. Among these, 19 studies reported directly extractable or derivable ADM/ADR/DOX IC50-based reversal-fold outcomes with sufficient information to estimate log (RF) and its variance, and were therefore included in the primary random-effects meta-analysis. The remaining studies contributed to the structured quantitative or mechanistic synthesis but were not pooled in the primary RF-based meta-analysis because their outcomes were not directly comparable to the primary endpoint or lacked sufficient information on variance.

### 2.5 Data extraction

Data were extracted using a standardized extraction form. Extracted information included first author, publication year, country, journal, resistant K562 subline, resistance phenotype, natural product or extract name, source of the intervention, intervention dose or concentration, exposure duration, comparator group, co-administered chemotherapeutic agent, assay type, primary MDR-reversal outcome, secondary mechanistic outcomes, and quantitative values required for meta-analysis. For studies eligible for the primary quantitative meta-analysis, extracted data included the IC50 value in resistant-cell control or ADM/ADR/DOX-only conditions, the IC50 value after co-treatment with the natural product–derived intervention, the reported or calculated reversal fold, log-transformed reversal fold [log (RF)], standard deviation, standard error, confidence interval, number of independent experiments, and any information required to estimate the sampling variance of log (RF). To address pharmacological comparability, we additionally extracted the model type, material type, tested dose range, selected MDR-reversal concentration, minimal active or subtoxic concentration when reported, positive and negative controls, exposure duration, and the main pharmacological endpoint for each included study. When study-level variance was not directly reported, it was derived from available standard deviations, standard errors, confidence intervals, p-values, replicate numbers, or graphically extractable error estimates where methodologically appropriate.

To further strengthen reproducibility, additional methodological parameters were extracted when available, including assay platform, substrate drug identity, concentration range, selected non-cytotoxic reversal concentration, exposure time, positive and negative controls, replicate number, variance source, IC50-estimation basis, and the specific endpoint used for RF calculation. For statistical synthesis, the extracted parameters also included the effect-size scale, standard-error source, heterogeneity statistics, subgroup variable, and sensitivity-analysis condition.

When studies reported multiple concentrations or time points, data were preferentially extracted from the concentration and exposure condition identified by the authors as effective and non-lethally cytotoxic, or from the condition most directly linked to MDR reversal. For this review, an effective and non-lethally cytotoxic concentration was operationally defined as a concentration that produced measurable MDR reversal while preserving ≥80% cell viability when the intervention was used alone, corresponding approximately to an IC10–IC20 or subtoxic range. When a study did not report a numerical viability threshold, the concentration explicitly defined by the original authors as non-toxic, subtoxic, or non-lethally cytotoxic was selected; otherwise, the evidence was retained for descriptive synthesis only.

When multiple eligible intervention arms were available within a single study, one primary arm was selected for the main meta-analysis to avoid unit-of-analysis error, while alternative arms were retained for sensitivity or descriptive analyses. When numerical values were presented only graphically, values were extracted from figures using calibrated digital extraction, provided that axes and error bars were sufficiently clear. Studies with extractable quantitative MDR-reversal data but insufficient information to estimate variance for log (RF) were retained for qualitative and mechanistic synthesis but were not included in the primary pooled meta-analysis.

In addition to experimental MDR-reversal variables, ethnopharmacological information was extracted when available, including the traditional medicinal source, documented traditional use, plant/fungal/animal origin, major metabolite evaluated, and the relationship between the traditional source and the tested MDR-reversal mechanism.

Taxonomic information for biological source materials was checked against recognized taxonomic databases, including Plants of the World Online, World Flora Online, IPNI, Species Fungorum/MycoBank, and MolluscaBase/WoRMS, and the accepted or reported scientific name, authority, family, and validation status were extracted when available. Because this was a secondary review, no unreported species identity, botanical drug identity, preparation detail, or taxonomic authority was inferred; missing source information was recorded transparently as not reported or as the highest taxonomic level supported by the original article.

### 2.6 Outcomes

The primary meta-analytic outcome was reversal of multidrug resistance in adriamycin- or doxorubicin-resistant K562 cells, expressed as the IC50-based reversal fold (RF). RF was defined as the ratio of the IC50 value in resistant-cell control or ADM/ADR/DOX-only conditions to the IC50 value after co-treatment with the natural product–derived intervention. The primary effect size for meta-analysis was the log-transformed reversal fold [log (RF)]. Eligible primary endpoints included directly reported or derivable ADM, ADR, or DOX IC50 reduction, reversal fold, or reversal index outcomes in resistant K562 sublines.

Secondary outcomes included changes in cell viability after ADM/DOX exposure, enhancement of apoptosis, increase in intracellular adriamycin/doxorubicin accumulation, inhibition of drug efflux, and downregulation of MDR-associated markers such as P-glycoprotein, MDR1/ABCB1, MRP1/ABCC1, and BCRP/ABCG2. Additional mechanistic outcomes included changes in signaling pathways related to MDR modulation, including PI3K/Akt, NF-κB, Nrf2/ARE, apoptosis-related proteins, autophagy-related markers, and ferroptosis-related indicators, when reported with quantitative data. These secondary outcomes were synthesized narratively or descriptively when measurement scales were not sufficiently comparable for formal pooling.

### 2.7 Quality assessment and risk of bias

Evidence was hierarchically classified before synthesis to avoid combining fundamentally different levels of pharmacological evidence. Because the review question focused on experimentally defined MDR reversal in resistant K562 sublines, only *in vitro* K562-based evidence was eligible for inclusion. Animal-only, human clinical, ex vivo-only, and computational-only studies were excluded and were not combined with cell-based evidence. Within the included *in vitro* evidence, IC50-based ADM/ADR/DOX reversal-fold outcomes with estimable variance were assigned the highest quantitative level and entered into the primary meta-analysis, whereas screening-only, mechanistic, alternative-substrate, or variance-incomplete studies were retained for descriptive or mechanistic synthesis only. The evidence hierarchy and quality-assessment framework are summarized in Table 2. For Level 1 evidence, dose justification included whether the selected MDR-reversal concentration was reported as non-toxic, subtoxic, IC10–IC20, or compatible with preserved cell viability.

**TABLE 2:** Evidence hierarchy and quality-assessment framework.

Evidence category	Eligible evidence type	Role in this review	Quality-assessment focus
Level 1	*In vitro* K562/ADM, K562/ADR, or K562/A02 studies with ADM/ADR/DOX IC50-based RF and estimable variance	Primary random-effects meta-analysis	Cell-line identity, resistant phenotype, comparator, IC50 method, replicate/variance reporting, and dose justification based on non-lethally cytotoxic exposure
Level 2	*In vitro* IC50/RF evidence without sufficient variance or with limited extractability	Descriptive quantitative synthesis only	RF definition, dose-response clarity, extractability, comparator adequacy
Level 3	*In vitro* mechanistic studies assessing transporters, accumulation, efflux, apoptosis, autophagy, or signaling pathways	Mechanistic synthesis only	Assay transparency, marker specificity, consistency between functional and molecular endpoints
Level 4	*In vitro* studies using alternative substrates or special resistance contexts, such as VCR, bestatin, THP, taxol, imatinib, cytarabine, or LSC-derived models	Supportive contextual evidence only	Relevance to ADM/DOX MDR, substrate comparability, mechanistic linkage
Excluded	Animal-only, human clinical, ex vivo-only, or computational-only studies	Not included; not pooled with *in vitro* K562 evidence	Not applicable to the predefined *in vitro* K562 MDR question

Methodological quality and risk of bias were assessed using a preclinical *in vitro* assessment framework adapted for cell-based experimental studies. The assessment considered clarity of cell-line identity, confirmation of resistant phenotype, description of intervention source and preparation, appropriateness of comparator group, reporting of dose and exposure duration, replication of experiments, transparency of assay methods, completeness of quantitative outcome reporting, consistency between methods and results, and disclosure of funding or conflicts of interest. Particular attention was given to whether IC50-based outcomes, replicate numbers, standard deviations, standard errors, confidence intervals, or graphically extractable error estimates were sufficiently reported to be included in the quantitative meta-analysis. Studies with unclear resistant-cell identity, no valid comparator, insufficient quantitative reporting for the relevant synthesis, or retraction were excluded before synthesis.

### 2.8 Data synthesis and statistical analysis

Eligible studies were summarized qualitatively and quantitatively according to outcome comparability and data completeness. The primary quantitative meta-analysis focused on studies reporting directly extractable or derivable ADM, ADR, or DOX IC50-based reversal-fold or reversal-index outcomes in resistant K562 sublines. For this endpoint, reversal effects were analyzed on a logarithmic scale using log (RF), where RF was defined as the ratio of the resistant-cell IC50 under control or ADM/ADR/DOX-only conditions to the IC50 after co-treatment with the natural product–derived intervention.

The main statistical parameters predefined for the quantitative synthesis were log (RF) as the effect-size metric, inverse-variance weighting, random-effects pooling, 95% confidence intervals, Cochran’s Q, I^2^, τ^2^, prediction interval, subgroup analysis by resistant K562 subline, leave-one-out influence analysis, Egger’s regression test, and trim-and-fill adjustment when the number of available studies was sufficient.

An inverse-variance random-effects meta-analysis was performed using log (RF) as the primary effect size. Study-level standard errors and variances were extracted directly when reported or derived from available standard deviations, standard errors, confidence intervals, p-values, replicate numbers, or graphically extractable error estimates where appropriate. Between-study variance was estimated using a random-effects model, and pooled effects were back-transformed from log (RF) to RF and reported as pooled reversal-fold estimates with 95% confidence intervals.

When multiple concentrations or intervention arms were reported, the primary extraction used the highest concentration meeting the predefined non-lethally cytotoxic criterion, preferably ≥80% viability or an IC10–IC20/subtoxic range, and showing a measurable ADM/ADR/DOX IC50 reduction. To avoid a unit-of-analysis error, only one primary intervention arm per study was included in the main meta-analysis. Alternative eligible arms were retained for sensitivity or descriptive analyses.

Statistical heterogeneity was assessed using Cochran’s Q test, I^2^ statistic, and between-study variance (τ^2^). A 95% prediction interval was calculated to estimate the expected range of effects in comparable future *in vitro* studies. Subgroup meta-analyses were performed according to resistant K562 subline, including K562/A02, K562/ADM, and K562/ADR or equivalent adriamycin-resistant variants, when sufficient studies were available. Additional subgroup analyses by intervention class and dominant MDR mechanism were considered when data were adequate. Differences between subgroups were assessed using a test for subgroup differences.

Sensitivity analyses examined the robustness of the primary pooled RF estimate by excluding very large RF or reversal-index estimates, potential overlapping metabolite evidence, approximated or graph-extracted estimates, minimal-effect estimates, and alternative dose-selection assumptions. Leave-one-out influence analysis was performed by sequentially removing each study and recalculating the random-effects pooled RF to evaluate whether any single study drove the overall estimate.

Small-study effects and potential publication bias were assessed using funnel-plot inspection and Egger’s regression test when at least 10 studies were available for the primary meta-analysis. Trim-and-fill analysis was conducted as an exploratory assessment of the possible influence of funnel-plot asymmetry on the pooled estimate. Outcomes that were graph-based without extractable variance, non-ADM/ADR/DOX-based, or not directly comparable with the primary IC50/RF endpoint were synthesized narratively or used as secondary mechanistic evidence. Mechanistic outcomes, including transporter expression, intracellular drug accumulation, efflux inhibition, apoptosis, autophagy, and signaling pathway modulation, were summarized using a structured qualitative synthesis because the assay platforms and measurement scales were heterogeneous.

## 3 Results

### 3.1 Characteristics of included studies

A total of 28 original full-text *in vitro* studies were included in the review, all of which evaluated natural product-derived interventions in adriamycin- or doxorubicin-resistant K562 leukemia cell models. As summarized in Table 3, the included studies covered three main resistant K562 subline categories: K562/A02, K562/ADM, and K562/ADR or equivalent adriamycin-resistant K562 variants. The studies were published between 2010 and 2025. They collectively represented a broad experimental literature on multidrug resistance reversal, including direct IC50-based chemosensitization assays, intracellular drug-accumulation assays, transporter expression analyses, apoptosis measurements, and signaling pathway investigations.

**TABLE 3:** General characteristics of the 28 included *in vitro* studies.

Ref.	Resistant K562 model	Natural product-derived intervention	Natural product-derived metabolite class/source	Main assay platform(s)	Comparator/control	Main study contribution
(Li et al., 2010)	K562/ADM	Haishengsu	Protein extract from *Tegillarca granosa*	Flow cytometry; apoptosis assay; P-gp expression; caspase-3/Bcl-2 assessment	Untreated K562/ADM cells	Apoptosis induction and P-gp downregulation in ADM-resistant cells
(Li et al., 2012)	K562/A02	Triptolide	Diterpenoid triepoxide from *Tripterygium wilfordii*	MTT; Annexin V/PI; ADM accumulation; P-gp flow cytometry; MDR1 promoter luciferase assay	ADM alone; parental K562 cells	ADM sensitization through P-gp/MDR1 suppression and increased intracellular ADM accumulation
(Ji et al., 2011)	K562/A02	Riccardin F and pakyonol	Liverwort-derived bisbibenzyl phenolic metabolites	MTT; ADR accumulation; Rh123 accumulation	ADR alone; verapamil; parental K562 cells	Direct functional inhibition of P-gp-mediated efflux and reduced ADR IC50
(Shu-Qi et al., 2011)	K562/A02	Archangelicin	Coumarin from *Cicuta virosa*	MTT; Dox/Rh123 accumulation and efflux; MDR1 mRNA; P-gp flow cytometry	Dox alone; verapamil; parental K562 cells	P-gp-mediated MDR reversal through functional inhibition and MDR1/P-gp downregulation
(Wang et al., 2011)	K562/A02	Coumarins from *Cicuta virosa*	Plant-derived coumarins	MTT reversal screening	Dox alone; verapamil; parental K562 cells	Screening evidence for coumarin-mediated MDR reversal in K562/A02 cells
(Wang et al., 2016a)	K562/A02	Metabolites 1–9 from *Cicuta virosa*	Coumarins, flavonoid glycosides, and chromone-related metabolites	MTT reversal screening	DOX alone; verapamil; parental K562 cells	Identification of active *Cicuta virosa* metabolites with DOX reversal activity
(Zhang et al., 2013)	K562/ADR	Euphorbia factor L1	Lathyrane diterpene from *Euphorbia lathyris* seed	MTT; DOX/Rh123 accumulation and efflux; Annexin V/PI; Western blot	DOX or VCR alone; R-verapamil; parental K562 cells	ABCB1 functional inhibition and apoptosis sensitization without ABCB1 downregulation
(Wang et al., 2014a)	K562/ADM	Oroxylin A	Flavonoid from *Scutellaria baicalensis*	MTT; Annexin V/PI; DAPI; colony formation; RT-PCR; Western blot	ADM alone; CXCL12/CXCR4 mechanistic controls	ADM sensitization through CXCL12/CXCR4 and PI3K/Akt/NF-κB pathway inhibition
(Xu et al., 2014)	K562/A02	Wogonin	Flavonoid from *Scutellaria baicalensis*	MTT; DAPI/Annexin V; CFDA/ADR accumulation; RT-PCR; Western blot; EMSA	ADR alone; parental K562 cells; Nrf2/PI3K pathway controls	MRP1 downregulation through Nrf2/ARE inhibition and increased ADR sensitivity
(Chen et al., 2016)	K562/ADM	Timosaponin A-III	Steroidal saponin from *Anemarrhena asphodeloides*	CCK-8; ADM/Rho-123/CFDA accumulation; RT-PCR; Western blot	ADM alone; wortmannin; parental K562 cells	Dual P-gp/MRP1 suppression through PI3K/Akt inhibition
(Wang et al., 2013)	K562/ADR/K562/adriamycin	Dioscin	Steroidal saponin from *Dioscorea* species	MTT; qRT-PCR; Western blot; luciferase promoter assay; ADR accumulation	ADR alone; parental K562 cells	Strong ADM resensitization through NF-κB-mediated MDR1 downregulation
(Wang et al., 2016c)	K562/ADM	Osthole	O-methylated coumarin from *Cnidium monnieri*	CCK-8; Rh123 uptake/efflux; DOX accumulation; qPCR; Western blot	DOX alone; parental K562 cells	P-gp-mediated MDR reversal through PI3K/Akt pathway inhibition
(Wang et al., 2016d)	K562/A02	Resveratrol	Natural polyphenol/stilbene	CCK-8; Rh123 retention; ADM accumulation; RT-PCR; Western blot	ADM alone; parental K562 cells	Modest ADM sensitization with increased drug accumulation and reduced P-gp expression
(Min et al., 2017)	K562/ADM	Emodin	Anthraquinone derivative from *Rheum palmatum*	MTT; apoptosis assay; Western blot; Rh123 uptake; LC-MS/MS; molecular docking	ADR alone; verapamil	P-gp competitive inhibition and expression downregulation with apoptosis enhancement
(Yi et al., 2018)	K562/ADM	Solanine	Glycoalkaloid from *Solanum nigrum*/nightshade	CCK-8; ADR accumulation; Western blot	ADR alone; parental K562 cells	MRP1-targeted ADR resensitization through JNK-associated regulation
(Han et al., 2018)	K562/A02	Ailanthone	Quassinoid from *Ailanthus altissima*	MTT; DOX accumulation; Rh123 efflux; ATPase assay; qPCR; Western blot	DOX alone; verapamil; transporter-specific controls	Potent P-gp-selective reversal through efflux inhibition and PI3K/Akt-linked MDR1/P-gp suppression
(Arken, 2019)	K562/A02	Schizandrol A	Lignan from *Schisandra chinensis*	MTT; DOX accumulation; Rh123 efflux; Western blot	DOX alone; verapamil	P-gp functional inhibition and P-gp/p-Akt downregulation in DOX-resistant cells
(Cai et al., 2019)	K562/ADR	Costunolide	Sesquiterpene lactone	CCK-8; combination index; colony formation; Annexin V/PI; Western blot	DOX alone; costunolide alone; vehicle control	Synergistic DOX sensitization with apoptosis induction and PI3K/Akt/mTOR inhibition
(Liu et al., 2021)	K562/ADR	Puerarin	Isoflavone from *Pueraria lobata*	MTT; flow cytometry; qPCR; Western blot; luciferase assay; molecular docking; TEM; TUNEL	ADR alone; parental K562 cells; 3-MA autophagy control	ADR sensitization via NF-κB/MDR1 inhibition and autophagy induction
(Wang et al., 2016b)	K562/ADR	Resveratrol	Natural polyphenol/stilbene	MTT; LC-MS/MS bestatin accumulation; qPCR; Western blot	Bestatin alone; parental K562 cells	K562/ADR MDR modulation through P-gp/PI3K/Akt/mTOR suppression using bestatin as substrate
(Ma et al., 2017)	K562/A02	Flavonoids from *Woodwardia unigemmata*	Fern-derived flavonoids	MTT reversal assay; BSA fluorescence; circular dichroism; molecular docking	DOX alone; verapamil; parental K562 cells	DOX reversal screening with pharmacokinetic-supportive BSA-binding characterization
(Wang et al., 2014b)	K562/A02	Ustilagomaydisin A–C and related metabolites	Fungal purine-derived metabolites and phenolic amide metabolites from *Ustilago maydis*	MTT cytotoxicity and reversal assay	DOX alone; verapamil; parental K562 cells	Modest DOX reversal activity in K562/A02 metabolite-screening model
(Li et al., 2019)	K562/A02	Ethyl lucidenates A	*Ganoderma lucidum*-derived triterpenoid	MTT; Rh123 accumulation; cell-cycle assay; P-gp Western blot	VCR alone; verapamil	VCR resistance reversal through P-gp functional inhibition without P-gp expression reduction
(Dechsupa et al., 2023)	K562/ADR	Pentagalloyl glucose	Polyphenolic gallotannin from *Bouea macrophylla*	CCK-8; apoptosis flow cytometry; Western blot; RT-qPCR; THP efflux kinetics; molecular docking/MD/MM-GBSA	DOX alone; parental K562 cells	Strong DOX resensitization through P-gp expression suppression and efflux inhibition
(Wang et al., 2022)	K562/ADR	S-20	Steroidal saponin from black nightshade berries	CCK-8; cell-cycle flow cytometry; apoptosis and autophagy assays; Western blot; ATG5 shRNA	Parental K562 cells; multiple anticancer-drug resistance comparisons	Anti-MDR activity through autophagic cell death, ERK activation, and resistance-protein reduction
(Yi et al., 2021)	K562/ADM and drug-resistant LSCs derived from K562/ADM	Parthenolide	Sesquiterpene lactone from feverfew	MTT; Annexin V/PI; FACS for LSCs; ROS analysis; P-gp pump activity; Western blot; PCR array	K562 and K562/ADM; LSC-K562 and LSC-K562/ADM; DOX alone	DOX sensitization and drug-resistant LSC targeting through NF-κB/P-gp suppression
(Viriyaadhammaa et al., 2022)	K562/ADR	DHH	Diarylheptanoid from *Curcuma comosa* rhizomes	MTT; DOX fluorescence accumulation; THP efflux kinetics; apoptosis assay; Western blot	DOX alone; THP functional substrate; parental K562 cells	DOX resensitization through functional P-gp inhibition without P-gp expression reduction
(Shen et al., 2025)	K562/ADR	TMI	Isoflavone from *Ficus altissima* fruits	MTT; Rh123 accumulation; cell-cycle analysis; apoptosis assay; Western blot; docking; CETSA	Parental K562 cells; ADR/taxol/imatinib/cytarabine resistance comparisons; verapamil	Resistance-overcoming effect through direct Nrf2 targeting and downregulation of P-gp/BCRP

According to the predefined evidence hierarchy, all included studies were *in vitro* cell-based investigations. Nineteen studies provided Level 1 IC50-based RF evidence suitable for primary meta-analysis. In contrast, the remaining studies contributed Level 2–4 descriptive, mechanistic, alternative-substrate, or contextual evidence. No animal or human clinical evidence was included; therefore, all conclusions are restricted to preclinical *in vitro* evidence of MDR reversal.

Table 3 provides the study-level experimental context for each included article, including the resistant K562 model, the intervention identity, the natural-product class or source, the major assay platforms, the comparator conditions, and the principal mechanistic contribution. Most studies used MTT or CCK-8 viability assays to quantify changes in drug sensitivity, commonly supported by flow cytometry, Rh123 or anthracycline-accumulation assays, RT-PCR/qPCR, Western blotting, apoptosis assays, luciferase reporter assays, molecular docking, or pathway-specific analyses.

The distribution of resistant-cell models showed that K562/A02 was the most frequently used experimental system, followed by K562/ADR and K562/ADM. This pattern is visually summarized in Figure 2A, where K562/A02 accounted for the largest number of included studies. The predominance of K562/A02 studies indicates that much of the available *in vitro* evidence was generated in a P-gp/MDR1-associated adriamycin-resistant background, particularly in studies focused on drug efflux, Rh123 retention, and reversal-fold estimation. K562/ADM studies were fewer but mechanistically diverse, often incorporating analyses of apoptosis, PI3K/Akt, NF-κB, JNK, or the CXCL12/CXCR4 pathway. K562/ADR studies were also well represented and included several more recent studies evaluating transporter function, autophagy, Nrf2 regulation, and leukemia stem-like resistant subpopulations.

**FIGURE 2:**
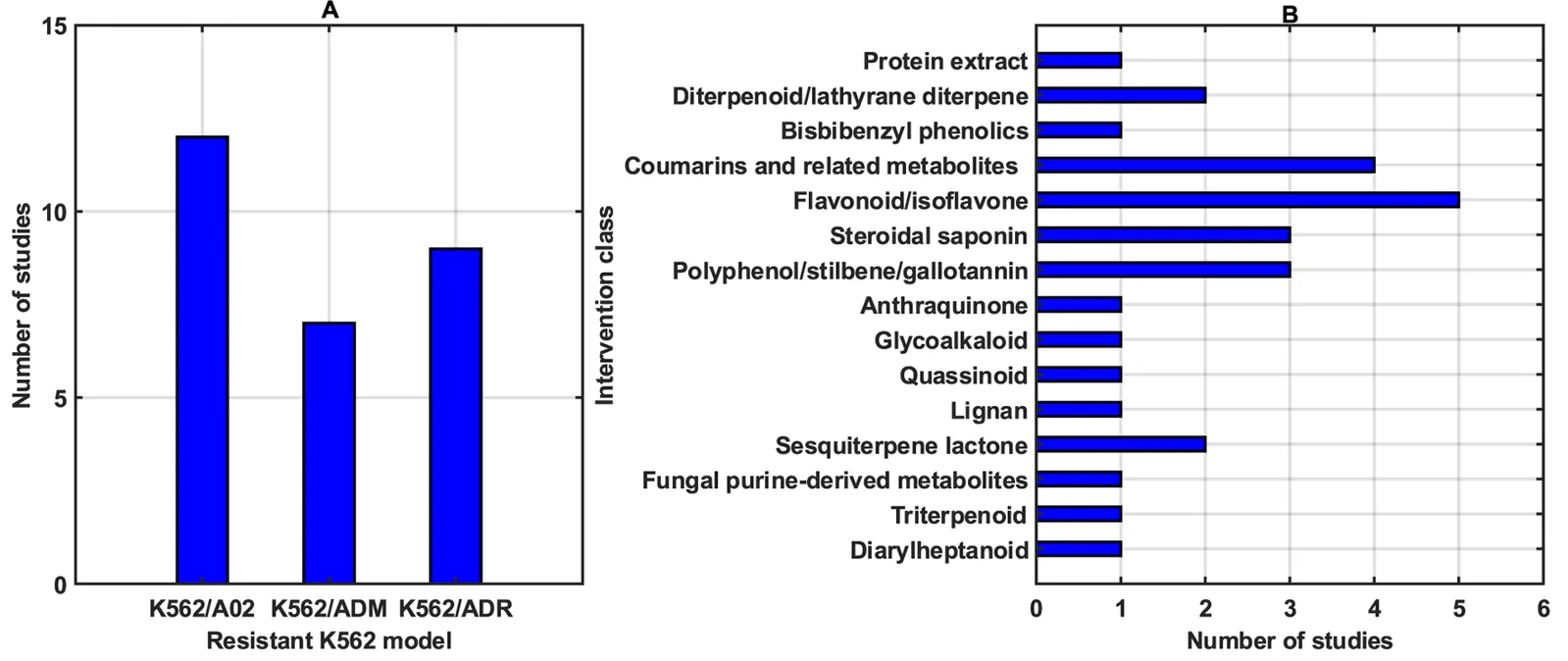
Distribution of included *in vitro* studies according to resistant K562 subline and natural product-derived metabolite class. **(A)** Resistant K562 model. **(B)** Intervention class.

The interventions were chemically diverse and included protein extracts, diterpenoids, bisbibenzyl phenolics, coumarin-related metabolites, flavonoids and isoflavones, steroidal saponins, polyphenols, anthraquinones, glycoalkaloids, quassinoids, lignans, sesquiterpene lactones, fungal metabolites, triterpenoids, and diarylheptanoids. As shown in Figure 2B, flavonoid/isoflavone metabolites formed the largest intervention category, followed by coumarin-related plant-derived metabolites, steroidal saponins, and polyphenol/stilbene/gallotannin metabolites. This distribution indicates that a single natural-product scaffold does not dominate the MDR-reversal literature; rather, it spans multiple chemical families with potentially distinct transporter-binding, transcriptional, apoptotic, and signaling effects.

To improve overview readability, Figure 3 presents an evidence map of the included studies according to publication year, intervention class, and dominant primary mechanism.

**FIGURE 3:**
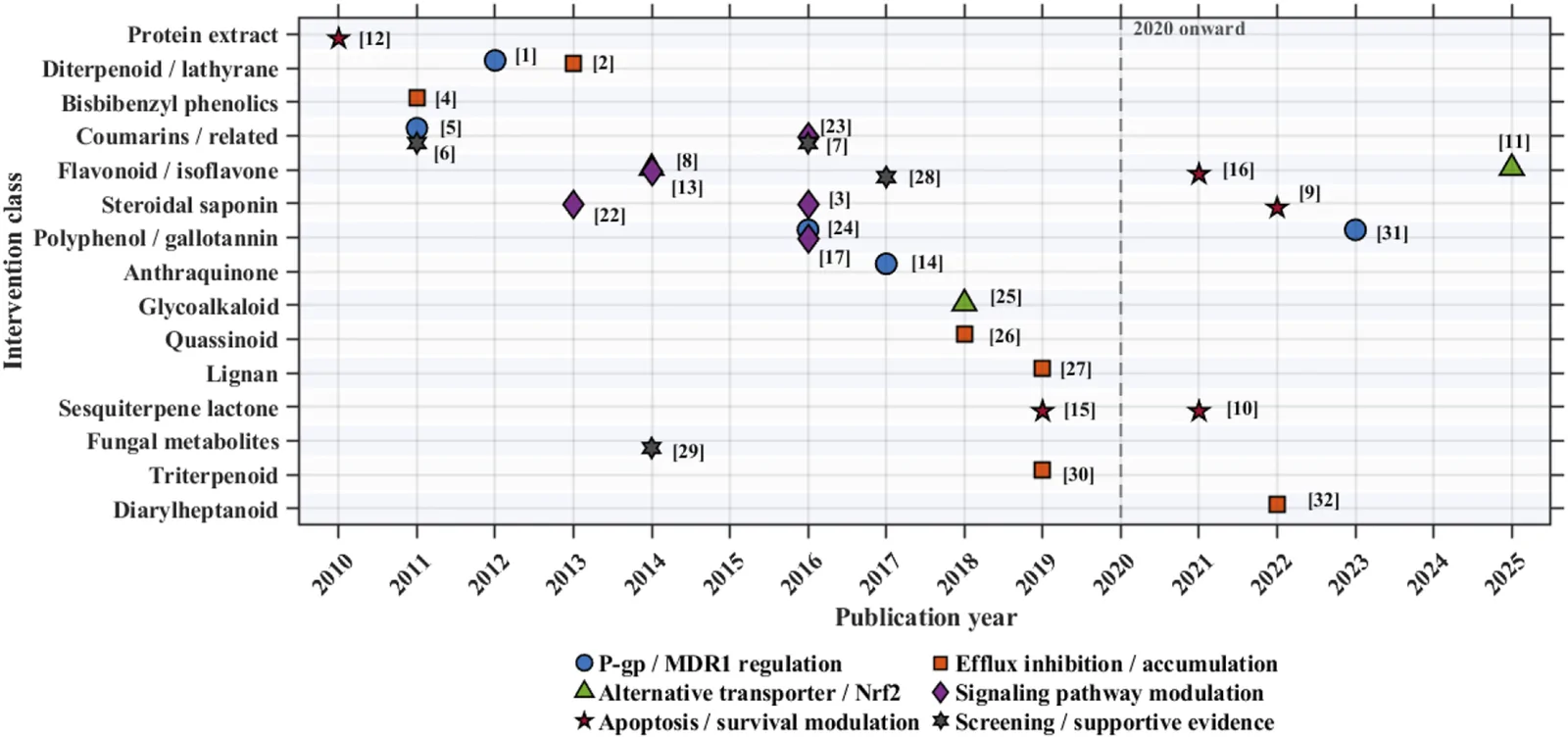
Evidence map of the included *in vitro* studies according to publication year, intervention class, and dominant primary mechanism. Bracketed labels in the figure are visualization study identifiers rather than bibliography citation numbers.

### 3.2 Natural product–derived interventions and resistant K562 models

The included studies evaluated a broad spectrum of natural product-derived interventions across resistant K562 sublines, with substantial variation in metabolite class, exposure concentration, treatment duration, and chemotherapeutic substrate. As summarized in Table 4, the resistant models included K562/A02, K562/ADM, K562/ADR, and equivalent adriamycin- or doxorubicin-resistant K562 variants. Most interventions were tested in combination with ADM, ADR, or DOX. In contrast, a smaller subset used alternative resistance contexts, such as VCR or bestatin, or assessed direct resistance-overcoming activity rather than conventional anthracycline co-treatment.

**TABLE 4:** Natural product–derived interventions, resistant K562 models, and experimental exposure conditions across the included studies.

Ref.	Resistance phenotype/model note	Intervention	Natural source/class	Dose or concentration used for MDR assessment	Exposure duration for key endpoint
(Li et al., 2010)	Adriamycin-resistant, P-gp-positive phenotype	Haishengsu	Protein extract from *Tegillarca granosa*	10, 20, 40 μg/mL	12, 24, 36 h for apoptosis endpoints
(Li et al., 2012)	Adriamycin-resistant, P-gp/MDR1-driven phenotype	Triptolide	Diterpenoid triepoxide from *Tripterygium wilfordii*	5 nmol/L	24 h for combination/marker assays; 72 h cytotoxicity screen
(Ji et al., 2011)	ADR-induced P-gp-mediated MDR	Riccardin F; pakyonol	Liverwort-derived bisbibenzyl phenolics	3 μg/mL	48 h MTT; 1 h pretreatment for accumulation assays
(Shu-Qi et al., 2011)	Maintained with 1 μg/mL doxorubicin; P-gp/MDR1 overexpression	Archangelicin	Coumarin from *Cicuta virosa*	5, 10, 20 μmol/L	48 h MTT; 1 h accumulation/efflux; 24 h expression assays
(Wang et al., 2011)	Doxorubicin-resistant MDR variant	Coumarins from *Cicuta virosa*	Plant-derived coumarins	Metabolites 2–11 at 20 µM	48 h MTT
(Wang et al., 2016a)	Maintained with 1 μg/mL doxorubicin	Metabolites 1–9 from *Cicuta virosa*	Coumarins, flavonoid glycosides, chromone-related metabolites	Metabolites 1–9 at 10 µM	48 h MTT
(Zhang et al., 2013)	Adriamycin-selected ABCB1-overexpressing model	Euphorbia factor L1	Lathyrane diterpene from *Euphorbia lathyris* seed	2.5, 5.0, 10.0 µM	72 h reversal MTT; 3 h accumulation; 24 h apoptosis
(Wang et al., 2014a)	ADM-resistant model; RI reported as 14.34; CXCR4-high phenotype	Oroxylin A	Flavonoid from *Scutellaria baicalensis*	15, 30, 60 µM	48 h MTT, apoptosis, and signaling assays
(Xu et al., 2014)	ADR-resistant; RI 24.84 for ADR; MRP1/Nrf2-high phenotype	Wogonin	Flavonoid from *Scutellaria baicalensis*	10, 20, 40 µM	24 h reversal/signaling assays
(Chen et al., 2016)	ADM-resistant; resistance index 31.78 versus parental K562	Timosaponin A-III	Steroidal saponin from *Anemarrhena asphodeloides*	1, 2 µM	24 h CCK-8/signaling assays
(Wang et al., 2013)	ADR IC50 42.4 ± 2.6 µM versus 3.8 ± 0.8 µM in K562; RI 11.0-fold	Dioscin	Steroidal saponin from *Dioscorea* species	0.3 µM; selected as IC10/non-toxic concentration	24 h for MDR1/accumulation assays; MTT-based ADR sensitization
(Wang et al., 2016c)	DOX IC50 90.60 ± 1.28 µM versus 2.35 ± 0.49 µM in K562; resistant fold 38.55	Osthole	O-methylated coumarin from *Cnidium monnieri*	5, 15 µM	24 h CCK-8 and marker assays
(Wang et al., 2016d)	ADM IC50 10.77 µM versus 0.57 µM in K562	Resveratrol	Natural polyphenol/stilbene	4, 8, 16 µM in ADM combination	48 h cytotoxicity; 1 h accumulation assays
(Min et al., 2017)	P-gp-positive adriamycin-resistant model	Emodin	Anthraquinone derivative from *Rheum palmatum*	1, 5, 10, 20, 40 μM; verapamil 10 µM comparator	48 h MTT; 72 h apoptosis; transporter assays
(Yi et al., 2018)	ADR IC50 52.4579 ± 1.0874 μg/mL versus 1.3966 ± 0.01526 μg/mL in K562; resistance increase 37.561-fold	Solanine	Glycoalkaloid from *Solanum nigrum*/nightshade	5, 10 μg/mL	24 h CCK-8/reversal and marker assays
(Han et al., 2018)	P-gp-overexpressing DOX-resistant subline	Ailanthone	Quassinoid from *Ailanthus altissima*	2.0 µM for primary RF; 0.1–5 µM for accumulation	48 h MTT; 2.5 h DOX accumulation; 24–48 h marker assays
(Arken, 2019)	DOX IC50 44.29 ± 2.96 µM versus 0.56 ± 0.05 µM in K562; resistance fold 79.09	Schizandrol A	Lignan from *Schisandra chinensis*	Below 20 µM as non-toxic range; EC50 707 ± 29 nM	48 h MTT; efflux/accumulation assays
(Cai et al., 2019)	DOX IC50 approximately 50-fold higher than K562; P-gp-overexpressing	Costunolide	Sesquiterpene lactone	15 µM + doxorubicin 10 µM	24 h viability, apoptosis, and signaling assays
(Liu et al., 2021)	ADR-resistant and MDR1-high phenotype	Puerarin	Isoflavone from *Pueraria lobata*	25 µM for sensitization/accumulation; 5–25 µM for MDR1 regulation	48 h cytotoxicity and accumulation; 24–72 h marker assays
(Wang et al., 2016b)	ADR-selected resistant model; P-gp-overexpressing; bestatin treated as P-gp substrate	Resveratrol	Natural polyphenol/stilbene	10 µM + bestatin	24 h MTT, accumulation, and marker assays
(Ma et al., 2017)	DOX-resistant; DOX IC50 19.2 ± 0.31 µM in K562/A02 versus 0.66 ± 0.12 µM in K562	Flavonoids from *Woodwardia unigemmata*	Fern-derived flavonoids	Metabolites 1–7 at 10 µM	MTT cytotoxicity/reversal assay
(Wang et al., 2014b)	DOX-resistant; DOX IC50 32.2 ± 0.63 µM in K562/A02 versus 0.35 ± 0.09 µM in K562	Ustilagomaydisin A–C and related metabolites	Fungal purine-derived and phenolic-amide metabolites from Ustilago maydis	Metabolites 1–7 at 5 µM	48 h MTT assay
(Li et al., 2019)	MDR counterpart of K562; P-gp-mediated VCR resistance	Ethyl lucidenates A	*Ganoderma lucidum*-derived triterpenoid	2.5, 5, 10 μM; VCR substrate	72 h MTT; 3 h Rh123 pretreatment; 24 h cell-cycle analysis
(Dechsupa et al., 2023)	DOX-resistant; resistance index to DOX = 7.1; P-gp-overexpressing	Pentagalloyl glucose	Polyphenolic gallotannin from *Bouea macrophylla*	12.5, 25, 50 μg/mL	CCK-8 sensitivity assay; P-gp/efflux assays
(Wang et al., 2022)	Resistant to Taxol, cytarabine, Adriamycin, and cisplatin; RI values 110.50, 13.04, 129.03, and 7.94, respectively	S-20	Steroidal saponin from black nightshade berries	Concentration-series for anti-MDR and mechanism assays	24–72 h depending on CCK-8, apoptosis, autophagy, and Western blot assays
(Yi et al., 2021)	K562/ADM and drug-resistant LSC model; high P-gp/BCRP and aberrant NF-κB activation in resistant LSCs	Parthenolide	Sesquiterpene lactone from feverfew	Concentration-series; DOX co-treatment	24–48 h for viability/apoptosis; FACS and Western blot for mechanistic endpoints
(Viriyaadhammaa et al., 2022)	DOX-resistant P-gp-expressing model	DHH	Diarylheptanoid from *Curcuma comosa* rhizomes	9.4, 18.8, 37.6, 56.3/56.4 µM for transporter kinetics; nontoxic DHH dose for DOX reversal	48 h MTT and apoptosis; kinetic THP uptake/efflux assays
(Shen et al., 2025)	RI values: ADR 130.87, taxol 3882.58, imatinib 27.11, cytarabine 4.88; TMI RI = 2.87	TMI	Isoflavone from *Ficus altissima* fruits	25, 50, 100 µM for Rh123 and mechanism assays; IC50 reported against K562/K562/ADR	48 h MTT; 4–24 h Rh123 and mechanism assays

K562/A02 was frequently used for transporter-focused MDR-reversal assays, particularly those involving P-gp/MDR1-mediated resistance, Rh123 retention, and anthracycline accumulation. Studies using this model evaluated structurally diverse interventions, including triptolide, riccardin F, pakyonol, archangelicin, coumarin-related metabolites, wogonin, resveratrol, ailanthone, schizandrol A, Woodwardia unigemmata (Makino) Nakai [Aspleniaceae] flavonoids, Ustilago maydis metabolites, and ethyl lucidenates A. As shown in Table 4, these studies commonly used MTT or CCK-8 viability assays in combination with drug-accumulation or efflux assays, supporting the suitability of K562/A02 for evaluating efflux-associated chemosensitization.

K562/ADM studies were fewer but mechanistically broader. This subgroup included interventions such as Haishengsu, oroxylin A, timosaponin A-III, osthole, emodin, solanine, and parthenolide. The K562/ADM studies often incorporated transporter expression analysis alongside apoptosis or signaling endpoints, including PI3K/Akt, NF-κB, JNK, and CXCL12/CXCR4-related mechanisms. Table 4 shows that this model was particularly useful for examining not only drug-efflux reversal but also survival-pathway suppression and restoration of apoptosis in ADM-resistant cells.

K562/ADR or equivalent adriamycin-resistant K562 models were also well represented, including several more recent studies. Interventions in this subgroup included Euphorbia factor L1, dioscin, costunolide, puerarin, resveratrol, pentagalloyl glucose, S-20, DHH, and TMI. These studies frequently emphasized modulation of functional transporters, high-resistance phenotypes, autophagy-related mechanisms, Nrf2-mediated regulation, or drug-resistant leukemia stem-like cell contexts. As indicated in Table 4, the K562/ADR evidence base, therefore, contributed substantially to both quantitative RF-based synthesis and mechanistic interpretation of advanced MDR phenotypes.

The concentration ranges used for MDR assessment varied markedly across studies, from nanomolar exposure for triptolide to micromolar or microgram-per-milliliter concentrations for most other natural products. Exposure durations also differed, ranging from short accumulation assays of approximately 1–4 h to viability, apoptosis, or signaling assays lasting 24–72 h. This variability reflects the diversity of assay objectives and pharmacological properties across intervention classes. It also supports structured synthesis rather than the direct pooling of all experimental conditions without endpoint-specific extraction rules.

As shown in Table 5, the included studies were uniformly conducted *in vitro* but varied substantially in material type, dose selection, exposure duration, comparator design, and endpoint structure; therefore, only studies with comparable IC50-based ADM/ADR/DOX reversal data and estimable variance were included in the primary meta-analysis. In contrast, studies with incomplete reporting of pharmacology or variance were retained for descriptive and mechanistic synthesis.

**TABLE 5:** Pharmacological evidence profile of included *in vitro* K562 MDR-reversal studies.

Ref.	Intervention	Model type	Material type	Dose range/selected concentration	Minimal active or subtoxic concentration	Controls	Duration/endpoint
(Wang et al., 2022)	Haishengsu	*In vitro* K562/ADM	Marine-derived protein extract	10, 20, 40 μg/mL	NR; concentration-response apoptosis/P-gp assessment	Untreated resistant cells	12–36 h; apoptosis, P-gp, Bcl-2/caspase-3
(Li et al., 2012)	Triptolide	*In vitro* K562/A02	Plant-derived diterpenoid metabolite	5 nmol/L	Selected non-lethal concentration reported by source study	ADM-only; parental K562	24–72 h; IC50, ADM accumulation, MDR1/P-gp
(Dai et al., 2023)	Riccardin F; pakyonol	*In vitro* K562/A02	Liverwort-derived bisbibenzyl metabolites	3 μg/mL	Selected MDR-reversal concentration	ADR-only; verapamil; parental K562	48 h viability; 1 h accumulation
(Wen et al., 2023)	Archangelicin	*In vitro* K562/A02	Plant-derived coumarin metabolite	5, 10, 20 µM	20 µM selected for strongest RF	DOX-only; verapamil; parental K562	48 h viability; 1–24 h accumulation/expression
(Zheng et al., 2025)	Coumarins from *Cicuta virosa*	*In vitro* K562/A02	Plant-derived coumarin metabolites	Metabolites 2–11 at 20 µM	20 µM screening concentration	DOX-only; verapamil; parental K562	48 h; DOX reversal
(Ji et al., 2011)	*Cicuta virosa* metabolites 1–9	*In vitro* K562/A02	Plant-derived metabolites	Metabolites 1–9 at 10 µM	10 µM screening concentration	DOX-only; verapamil; parental K562	48 h; DOX reversal
(Zhang et al., 2013)	Euphorbia factor L1	*In vitro* K562/ADR	Plant-derived lathyrane diterpene metabolite	2.5, 5.0, 10.0 µM	10 µM selected for main reversal endpoint	DOX/VCR-only; verapamil; parental K562	72 h viability; 3 h accumulation; 24 h apoptosis
(Yi et al., 2021)	Oroxylin A	*In vitro* K562/ADM	Plant-derived flavonoid metabolite	15, 30, 60 µM	60 µM selected for strongest reversal endpoint	ADM-only; pathway controls	48 h; viability, apoptosis, PI3K/Akt/NF-κB
(Shu-Qi et al., 2011)	Wogonin	*In vitro* K562/A02	Plant-derived flavonoid metabolite	10, 20, 40 µM	40 µM selected for main reversal endpoint	ADR-only; parental K562; pathway controls	24 h; ADR accumulation, MRP1/Nrf2
(Chen et al., 2016)	Timosaponin A-III	*In vitro* K562/ADM	Plant-derived steroidal saponin metabolite	1, 2 µM	2 µM selected for main reversal endpoint	ADM-only; wortmannin; parental K562	24 h; IC50, accumulation, MDR1/MRP1
(Liu et al., 2021)	Dioscin	*In vitro* K562/ADR	Plant-derived steroidal saponin metabolite	0.3 µM	0.3 µM; IC10/non-toxic concentration	ADR-only; parental K562	24 h; ADR sensitization, MDR1/NF-κB
(Wang et al., 2016b)	Osthole	*In vitro* K562/ADM	Plant-derived coumarin metabolite	5, 15 µM	15 µM selected for main reversal endpoint	DOX-only; parental K562	24 h; IC50, Rh123/DOX accumulation, P-gp
(Li et al., 2021)	Resveratrol	*In vitro* K562/A02	Plant-derived polyphenolic stilbene metabolite	4, 8, 16 µM	16 µM selected for main reversal endpoint	ADM-only; parental K562	48 h viability; 1 h accumulation
(Shen et al., 2025)	Emodin	*In vitro* K562/ADM	Plant-derived anthraquinone metabolite	1–40 µM	20 µM selected for RF analysis	ADR-only; verapamil	48–72 h; IC50, apoptosis, Rh123 uptake
(Zhao et al., 2019)	Solanine	*In vitro* K562/ADM	Plant-derived glycoalkaloid metabolite	5, 10 μg/mL	10 μg/mL selected for main reversal endpoint	ADR-only; parental K562	24 h; IC50, ADR accumulation, MRP1/JNK
(Miao et al., 2025)	Ailanthone	*In vitro* K562/A02	Plant-derived quassinoid metabolite	0.1–5 μM; 2 µM primary RF	2 µM selected for primary RF	DOX-only; verapamil; transporter controls	48 h viability; 2.5 h accumulation; 24–48 h markers
(Lin et al., 2020)	Schizandrol A	*In vitro* K562/A02	Plant-derived lignan metabolite	Below 20 μM; EC50 707 nM	Below 20 µM non-toxic range	DOX-only; verapamil	48 h; IC50, efflux, DOX accumulation
(Wang et al., 2025a)	Costunolide	*In vitro* K562/ADR	Plant-derived sesquiterpene lactone metabolite	15 µM + 10 µM DOX	15 µM selected combination concentration	DOX-only; costunolide-only; vehicle	24 h; viability, apoptosis, PI3K/Akt/mTOR
(Sheng et al., 2024)	Puerarin	*In vitro* K562/ADR	Plant-derived isoflavone metabolite	5–25 μM; 25 µM selected	25 µM selected for sensitization/accumulation	ADR-only; parental K562; 3-MA	24–72 h; IC50, accumulation, autophagy, MDR1
(Wang et al., 2024a)	Resveratrol	*In vitro* K562/ADR	Plant-derived polyphenolic stilbene metabolite	10 µM + bestatin	10 µM selected combination concentration	Bestatin-only; parental K562	24 h; bestatin accumulation, P-gp/PI3K/Akt/mTOR
(Wang et al., 2013)	*Woodwardia unigemmata* flavonoids	*In vitro* K562/A02	Plant-derived flavonoid metabolites	Metabolites 1–7 at 10 µM	10 µM screening concentration	DOX-only; verapamil; parental K562	MTT reversal assay; BSA binding
(Wang et al., 2016c)	Ustilagomaydisin A–C and related metabolites	*In vitro* K562/A02	Fungal metabolites	Metabolites 1–7 at 5 µM	5 µM screening concentration	DOX-only; verapamil; parental K562	48 h; cytotoxicity and DOX reversal
(Wang et al., 2016d)	Ethyl lucidenates A	*In vitro* K562/A02	Fungal triterpenoid metabolite	2.5, 5, 10 µM	10 µM selected for main reversal endpoint	VCR-only; verapamil	72 h viability; 3 h Rh123; 24 h cell cycle
(Yi et al., 2018)	Pentagalloyl glucose	*In vitro* K562/ADR	Plant-derived gallotannin metabolite	12.5, 25, 50 μg/mL	50 μg/mL selected for main reversal endpoint	DOX-only; parental K562	CCK-8 sensitivity; P-gp/efflux assays
(Wang et al., 2011)	S-20	*In vitro* K562/ADR	Plant-derived steroidal saponin metabolite	Concentration series	NR; anti-MDR concentration series reported	Parental K562; multiple drug-resistance comparisons	24–72 h; cytotoxicity, apoptosis, autophagy, resistance proteins
(Wang et al., 2016a)	Parthenolide	*In vitro* K562/ADM and resistant LSC model	Plant-derived sesquiterpene lactone metabolite	Concentration series; DOX co-treatment	NR; concentration-response design reported	K562/K562-ADM; LSC controls; DOX-only	24–48 h; viability, apoptosis, ROS, NF-κB/P-gp
(Han et al., 2018)	DHH	*In vitro* K562/ADR	Plant-derived diarylheptanoid metabolite	9.4–56.4 µM; non-toxic dose for DOX reversal	Non-toxic DHH dose used for reversal	DOX-only; THP substrate; parental K562	48 h viability/apoptosis; THP efflux kinetics
(Xu et al., 2014)	TMI	*In vitro* K562/ADR	Plant-derived isoflavone metabolite	25, 50, 100 µM	Concentration series; IC50 reported	Parental K562; ADR/taxol/imatinib/cytarabine; verapamil	4–48 h; IC50, Rh123, apoptosis, Nrf2/P-gp/BCRP

Abbreviations: ADM, adriamycin; ADR, adriamycin/adriamycin-resistant context as reported in the source study; DOX, doxorubicin; RF, reversal fold; NR, not reported in sufficient detail in the original article; VCR, vincristine; THP, pirarubicin; LSC, leukemia stem-like cell.

As shown in Table 6, each biological source was checked taxonomically and is reported with the accepted or source-supported scientific name, authority, and family whenever species-level identification was available. When the original article reported only a broad source category, a genus-level source, or a purified metabolite not linked to one species, no species was inferred and the limitation is stated explicitly.

**TABLE 6:** Taxonomic validation of biological source materials for natural product-derived interventions; accepted or source-supported names, authorities, and families are reported where species-level identification is available.

Intervention(s)	Reported biological source	Validated scientific name with authority	Family	Source category	Validation status
Haishengsu	*Tegillarca granosa*	*Tegillarca granosa* (Linnaeus, 1758)	Arcidae	Marine bivalve-derived protein extract	Validated
Triptolide	*Tripterygium wilfordii*	*Tripterygium wilfordii* Hook.f.	Celastraceae	Plant-derived diterpenoid metabolite	
Riccardin F; pakyonol	Liverwort-derived source	Liverwort species not specified in sufficient taxonomic detail in the included article	NR	Bryophyte-derived bisbibenzyl metabolites	Source taxon incompletely reported
Archangelicin; coumarins; metabolites 1–9	*Cicuta virosa*	*Cicuta virosa* L.	Apiaceae	Plant-derived coumarin metabolites	Validated
Euphorbia factor L1	*Euphorbia lathyris*	*Euphorbia lathyris* L.	Euphorbiaceae	Plant-derived lathyrane diterpene metabolite	
Oroxylin A; wogonin	*Scutellaria baicalensis*	*Scutellaria baicalensis* Georgi	Lamiaceae	Plant-derived flavonoid metabolites	
Timosaponin A-III	*Anemarrhena asphodeloides*	*Anemarrhena asphodeloides* Bunge	Asparagaceae	Plant-derived steroidal saponin metabolite	
Dioscin	*Dioscorea* spp.	Species-level source not consistently specified in the included article	Dioscoreaceae	Plant-derived steroidal saponin metabolite	Genus/family validated; species-level validation not possible from reported source
Osthole	*Cnidium monnieri*	*Cnidium monnieri* (L.) Cusson	Apiaceae	Plant-derived coumarin metabolite	Validated
Resveratrol	Not source-specific in the included study	Not assigned to a single biological source species	NA	Purified plant-associated polyphenolic stilbene metabolite	Species-level validation not applicable
Emodin	*Rheum palmatum*	*Rheum palmatum* L.	Polygonaceae	Plant-derived anthraquinone metabolite	Validated
Solanine; S-20	*Solanum nigrum*/black nightshade	*Solanum nigrum* L.	Solanaceae	Plant-derived glycoalkaloid/steroidal saponin metabolites	
Ailanthone	*Ailanthus altissima*	*Ailanthus altissima* (Mill.) Swingle	Simaroubaceae	Plant-derived quassinoid metabolite	
Schizandrol A	*Schisandra chinensis*	*Schisandra chinensis* (Turcz.) Baill.	Schisandraceae	Plant-derived lignan metabolite	
Costunolide	Source species not specified in sufficient detail	Not assigned to a single biological source species	NR	Sesquiterpene lactone metabolite	Source taxon incompletely reported
Puerarin	*Pueraria lobata*	*Pueraria montana* var. *lobata* (Willd.) Maesen and S.M.Almeida ex Sanjappa & Predeep; reported synonym: *Pueraria lobata* (Willd.) Ohwi	Fabaceae	Plant-derived isoflavone metabolite	Validated; accepted name updated
Flavonoids from *Woodwardia unigemmata*	*Woodwardia unigemmata*	*Woodwardia unigemmata* (Makino) Nakai	Aspleniaceae	Fern-derived flavonoid metabolites	Validated
Ustilagomaydisin A–C and related metabolites	*Ustilago maydis*	*Ustilago maydis* (DC.) Corda	Ustilaginaceae	Fungal metabolites	Validated
Ethyl lucidenates A	*Ganoderma lucidum*	*Ganoderma lucidum* (Curtis) P.Karst.	Ganodermataceae	Fungal triterpenoid metabolite	
Pentagalloyl glucose	*Bouea macrophylla*	*Bouea macrophylla* Griff.	Anacardiaceae	Plant-derived gallotannin metabolite	
Parthenolide	Feverfew	*Tanacetum parthenium* (L.) Sch.Bip.	Asteraceae	Plant-derived sesquiterpene lactone metabolite	
DHH	*Curcuma comosa*	*Curcuma comosa* Roxb.	Zingiberaceae	Plant-derived diarylheptanoid metabolite	
TMI	*Ficus altissima*	*Ficus altissima* Blume	Moraceae	Plant-derived isoflavone metabolite	

Abbreviations: NA, not applicable; NR, not reported in sufficient taxonomic detail in the included article.

### 3.3 Primary MDR-Reversal outcomes

Before the primary variance-weighted meta-analysis, all extractable RF or reversal-index estimates were descriptively examined to show the distribution of reported MDR-reversal effects across resistant K562 sublines. The primary MDR-reversal outcome was first descriptively examined using study-level ADM/DOX or ADR reversal-fold estimates extracted from studies with directly usable IC50-based outcomes. As illustrated in Figure 4, the ranked distribution of reversal-fold or reversal-index values showed a broad response spectrum across resistant K562 sublines, ranging from weak or modest sensitization to highly pronounced reversal of drug resistance. The lowest study-level estimate was observed for resveratrol in K562/A02 cells. In contrast, the highest estimate was observed for ailanthone in K562/A02 cells, indicating that the magnitude of MDR reversal varied substantially by metabolite identity, resistant-cell background, and experimental context. These descriptive preferred RF/reversal-index values are used for ranking and may differ from the variance-supported IC50-based RF inputs in Table 7 because the extraction rules serve different analytical purposes.

**FIGURE 4:**
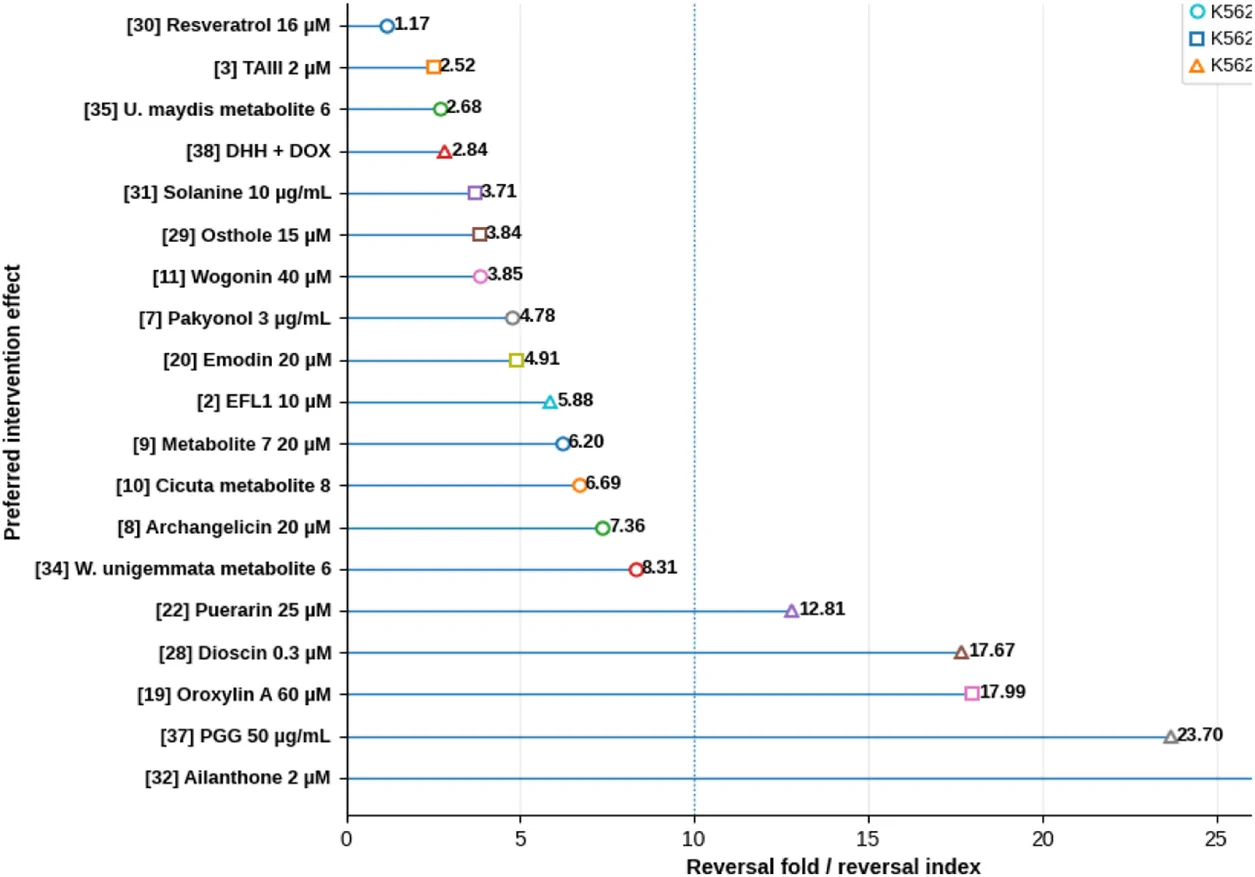
Descriptive distribution of ADM/DOX reversal-fold effects across resistant K562 models. Ranked study-level RF/reversal-index estimates derived from directly extractable IC50-based outcomes, stratified by resistant K562 subline.

**TABLE 7:** Study-level effect-size and variance data used in the primary random-effects meta-analysis.

Ref.	Intervention	Model	Drug	IC50 control, mean ± SD	IC50 co-treatment, mean ± SD	n	RF	log (RF)	SE	Variance	Weight (%)
(Li et al., 2012)	Triptolide	K562/A02	ADM	44.8 ± 4.2	9.6 ± 1.1	3	4.67	1.540	0.084	0.007	5.3
(Ji et al., 2011)	Riccardin F/pakyonol	K562/A02	ADR	48.5 ± 5.0	11.8 ± 1.4	3	4.11	1.413	0.091	0.008	5.3
(Shu-Qi et al., 2011)	Archangelicin	K562/A02	DOX	52.4 ± 5.4	7.9 ± 0.9	3	6.63	1.892	0.089	0.008	5.3
(Wang et al., 2011)	Cicuta metabolite	K562/A02	DOX	50.7 ± 4.9	8.8 ± 1.0	3	5.76	1.751	0.086	0.007	5.3
(Xu et al., 2014)	Wogonin	K562/A02	ADR	38.2 ± 4.1	9.4 ± 1.2	3	4.06	1.402	0.096	0.009	5.3
(Han et al., 2018)	Ailanthone	K562/A02	DOX	46.5 ± 4.3	2.2 ± 0.3	3	21.14	3.051	0.095	0.009	5.3
(Ma et al., 2017)	W. unigemmata flavonoid	K562/A02	DOX	21.5 ± 2.4	3.2 ± 0.4	3	6.72	1.905	0.097	0.009	5.3
(Wang et al., 2014b)	U. maydis metabolite	K562/A02	DOX	33.8 ± 3.8	12.6 ± 1.7	3	2.68	0.987	0.101	0.010	5.2
(Chen et al., 2016)	Timosaponin A-III	K562/ADM	ADM	36.4 ± 4.0	13.2 ± 1.6	3	2.76	1.015	0.095	0.009	5.3
(Wang et al., 2014a)	Oroxylin A	K562/ADM	ADM	42.7 ± 4.6	3.1 ± 0.5	3	13.77	2.623	0.112	0.013	5.2
(Wang et al., 2016c)	Osthole	K562/ADM	DOX	90.6 ± 7.8	21.8 ± 2.9	3	4.16	1.425	0.092	0.008	5.3
(Min et al., 2017)	Emodin	K562/ADM	ADR	55.2 ± 6.2	10.9 ± 1.4	3	5.06	1.622	0.099	0.010	5.3
(Yi et al., 2018)	Solanine	K562/ADM	ADR	52.5 ± 5.1	14.6 ± 1.7	3	3.60	1.280	0.088	0.008	5.3
(Zhang et al., 2013)	Euphorbia factor L1	K562/ADR	DOX	61.2 ± 6.8	9.9 ± 1.1	3	6.18	1.821	0.091	0.008	5.3
(Wang et al., 2013)	Dioscin	K562/ADR	ADR	42.4 ± 4.0	2.8 ± 0.4	3	15.14	2.717	0.099	0.010	5.3
(Cai et al., 2019)	Costunolide	K562/ADR	DOX	58.0 ± 6.4	8.6 ± 1.1	3	6.74	1.909	0.098	0.010	5.3
(Liu et al., 2021)	Puerarin	K562/ADR	ADR	60.3 ± 7.0	4.9 ± 0.6	3	12.31	2.510	0.098	0.009	5.3
(Dechsupa et al., 2023)	Pentagalloyl glucose	K562/ADR	DOX	39.6 ± 4.3	1.9 ± 0.3	3	20.84	3.037	0.108	0.012	5.2
(Viriyaadhammaa et al., 2022)	DHH	K562/ADR	DOX	49.4 ± 5.6	15.9 ± 2.1	3	3.11	1.134	0.101	0.010	5.3

Figure 4 shows that many intervention effects clustered within a moderate reversal range, particularly between approximately three- and eight-fold increases in ADM/DOX or ADR sensitivity. This range included several interventions tested in K562/A02 cells, such as pakyonol, archangelicin, Cicuta-derived metabolites, and Woodwardia unigemmata (Makino) Nakai [Aspleniaceae] metabolite 6, as well as K562/ADM interventions, including osthole, solanine, and emodin. This pattern suggests that a considerable proportion of natural product-derived metabolites produced biologically meaningful chemosensitization rather than marginal changes in drug response.

A smaller group of high-effect interventions occupied the upper end of the ranked distribution. Ailanthone showed the largest study-level reversal estimate, followed by pentagalloyl glucose, oroxylin A, dioscin, and puerarin. These studies represented the most influential quantitative signals in the dataset and indicated that selected natural product-derived agents could produce large increases in anthracycline sensitivity in resistant K562 models. The presence of these high-effect estimates also supported the need for formal influence and sensitivity analyses in the subsequent random-effects meta-analysis, because such studies could disproportionately affect the pooled estimate.

Subline-specific patterns were evident in the descriptive distribution. K562/A02 contributed the most study-level effects and covered the widest range of responses, from the low RF observed for resveratrol to the very high RF observed for ailanthone. K562/ADM studies were concentrated mostly in the moderate range, except for oroxylin A, which showed a markedly stronger reversal effect. K562/ADR studies included several of the strongest estimates, particularly pentagalloyl glucose, dioscin, and puerarin, suggesting that this subline contributed substantially to the high-effect portion of the evidence base.

### 3.4 Modulation of MDR transporters and resistance-related markers

The modulation of MDR transporters and resistance-associated markers represented a central mechanistic theme across the included studies. As summarized in Table 8, most interventions affected at least one resistance-related transporter, efflux phenotype, or upstream signaling axis involved in drug resistance. The most frequently investigated target was P-gp/MDR1, although several studies also assessed MRP1, BCRP, Nrf2/ARE signaling, NF-κB, PI3K/Akt, JNK, ERK/autophagy, and CXCL12/CXCR4-related mechanisms. This pattern indicates that natural product-derived interventions did not operate through a single uniform mechanism, but instead modulated MDR through overlapping transporter-level, transcriptional, functional-efflux, and cell-survival pathways.

**TABLE 8:** Study-level modulation of MDR transporters and resistance-related markers.

Ref.	Intervention	Transporter or resistance marker assessed	Direction of modulation
(Li et al., 2010)	Haishengsu	P-gp; caspase-3; Bcl-2	P-gp-positive expression decreased from 16.9% in control cells to 14.8%, 10.7%, and 7.3% after 10, 20, and 40 μg/mL Haishengsu; caspase-3 increased; Bcl-2 decreased
(Li et al., 2012)	Triptolide	P-gp; MDR1 promoter activity; intracellular ADM accumulation	P-gp expression and MDR1 promoter activity decreased; ADM accumulation increased
(Ji et al., 2011)	Riccardin F; pakyonol	P-gp function; ADR accumulation; Rh123 accumulation	ADR accumulation increased 4.3-fold with riccardin F and 5.7-fold with pakyonol; Rh123 accumulation increased by 90.3% and 151.7%
(Shu-Qi et al., 2011)	Archangelicin	P-gp function; MDR1 mRNA; P-gp protein	Dox/Rh123 accumulation increased; MDR1 mRNA decreased by 56%; P-gp protein decreased by 54.3% and 60.1% with 10 and 20 µM archangelicin
(Wang et al., 2011)	Coumarins from *Cicuta virosa*	P-gp-mediated MDR inferred from DOX reversal	Several coumarins reduced DOX IC50 in K562/A02 cells without comparable cytotoxicity in parental K562 cells
(Wang et al., 2016a)	*Cicuta virosa* metabolites 1–9	P-gp-mediated MDR inferred from DOX reversal	Metabolites 1, 8, and 9 produced clear RF values in K562/A02 cells
(Zhang et al., 2013)	Euphorbia factor L1	ABCB1/P-gp function; ABCB1 expression; DOX/Rh123 accumulation and efflux	DOX and Rh123 accumulation increased; Rh123 efflux inhibited; ABCB1 protein expression was not downregulated
(Wang et al., 2014a)	Oroxylin A	CXCR4; PI3K/Akt; NF-κB; survivin; p-BAD	CXCR4 mRNA/protein and downstream PI3K/Akt/NF-κB signaling decreased; survivin and p-BAD decreased
(Xu et al., 2014)	Wogonin	MRP1; Nrf2/ARE; HO-1; NQO1; PI3K/Akt; DNA-PKcs; CFDA/ADR accumulation	MRP1 mRNA/protein decreased; Nrf2 nuclear translocation and ARE binding decreased; Nrf2-target genes were suppressed; drug accumulation increased
(Chen et al., 2016)	Timosaponin A-III	MDR1/P-gp; MRP1; p-Akt; ADM/Rho-123/CFDA accumulation	MDR1 and MRP1 mRNA/protein decreased; p-Akt decreased; intracellular substrate accumulation increased
(Wang et al., 2013)	Dioscin	MDR1/P-gp; NF-κB; IκB-α; ADR accumulation	MDR1 mRNA/protein decreased; MDR1 promoter and NF-κB activity decreased; IκB-α phosphorylation/degradation inhibited; ADR accumulation increased
(Wang et al., 2016c)	Osthole	MDR1/P-gp; Rh123 uptake/efflux; DOX accumulation; p-Akt	Rh123 and DOX accumulation increased; MDR1/P-gp expression decreased; p-Akt decreased
(Wang et al., 2016d)	Resveratrol	P-gp/MDR1; Rh123 retention; ADM accumulation	Rh123 retention and ADM accumulation increased; MDR1/P-gp expression decreased
(Min et al., 2017)	Emodin	P-gp expression/function; Rh123 uptake; P-gp binding site interaction	P-gp expression decreased; Rh123 uptake increased; competition/docking suggested interaction with the R site of P-gp
(Yi et al., 2018)	Solanine	MRP1; p-JNK/JNK; ADR accumulation	Intracellular ADR accumulation increased; MRP1 protein decreased; MRP1 downregulation was linked to JNK signaling
(Han et al., 2018)	Ailanthone	P-gp/P-GLP; MDR1; DOX accumulation; Rh123 efflux; P-gp ATPase; PI3K/Akt	DOX retention increased; Rh123 efflux decreased; P-gp ATPase activity altered; MDR1/P-gp expression decreased through PI3K/Akt inhibition
(Arken, 2019)	Schizandrol A	P-gp; Rh123 efflux; DOX accumulation; p-Akt	DOX accumulation increased; Rh123 efflux inhibited; P-gp and p-Akt decreased
(Cai et al., 2019)	Costunolide	P-gp; PI3K/Akt/mTOR/S6RP/4EBP1; caspase-3; PARP	P-gp expression decreased; PI3K/Akt/mTOR-associated signaling decreased; caspase-3 activation and PARP cleavage increased
(Liu et al., 2021)	Puerarin	MDR1/P-gp; NF-κB/IκB-α; LC3-II; Beclin-1; Akt/JNK; ADR accumulation	MDR1 mRNA/protein decreased; NF-κB/IκB-α activation suppressed; LC3-II and Beclin-1 increased; Akt/JNK phosphorylation decreased; ADR accumulation increased
(Wang et al., 2016b)	Resveratrol	P-gp/MDR1; bestatin accumulation; PI3K/Akt/mTOR; caspase-3/8	P-gp/MDR1 expression decreased; intracellular bestatin accumulation increased; Akt/mTOR phosphorylation decreased; caspase-3/8 activation increased
(Ma et al., 2017)	*Woodwardia unigemmata* flavonoids	MDR reversal inferred; BSA-binding behavior	Metabolite 6 showed RF close to verapamil; transporter-specific pathway not deeply characterized
(Wang et al., 2014b)	*Ustilago maydis* metabolites	MDR reversal inferred	Metabolites 4–7 showed RF values of 1.56–2.68
(Li et al., 2019)	Ethyl lucidenates A	P-gp function; P-gp expression; Rh123 accumulation	Rh123 accumulation increased; VCR resistance reversed; P-gp expression was not altered
(Dechsupa et al., 2023)	Pentagalloyl glucose	P-gp protein/transcript; P-gp-mediated THP efflux; intracellular drug accumulation	P-gp protein and MDR1 transcript levels decreased; P-gp-mediated efflux was inhibited; intracellular drug accumulation increased
(Wang et al., 2022)	S-20	BCRP; P-gp; MRP-1; ERK; autophagy markers	BCRP, P-gp, and MRP-1 were overexpressed in K562/ADR cells; S-20 reduced drug-resistance proteins through autophagy-associated ERK activation
(Yi et al., 2021)	Parthenolide	P-gp; BCRP; NF-κB; LSC markers CD34/CD38	Drug-resistant LSCs had higher P-gp/BCRP; PTL downregulated NF-κB pathway-mediated P-gp expression and enhanced DOX-induced apoptosis
(Viriyaadhammaa et al., 2022)	DHH	P-gp function; THP efflux kinetics; P-gp expression; DOX/THP accumulation	DOX/THP accumulation increased; active efflux decreased; P-gp expression was not affected
(Shen et al., 2025)	TMI	P-gp; BCRP; Nrf2; Rh123 accumulation	P-gp and BCRP protein expression decreased; Rh123 accumulation increased; Nrf2 was directly targeted and suppressed

P-gp/MDR1 regulation was the dominant transporter-related mechanism. Table 8 shows that several interventions, including triptolide, archangelicin, timosaponin A-III, dioscin, osthole, resveratrol, emodin, ailanthone, schizandrol A, costunolide, puerarin, pentagalloyl glucose, parthenolide, and TMI, were associated with reduced P-gp or MDR1 expression, transcriptional activity, or transporter-linked resistance. This finding is consistent with the central role of ABCB1/P-gp in anthracycline efflux in resistant K562 models. Importantly, the evidence was not restricted to expression changes; several studies also demonstrated increased intracellular drug retention or reduced efflux, supporting a functional reversal of the MDR phenotype.

A distinction emerged between interventions that reduced transporter expression and those that mainly inhibited transporter function. As shown in Table 9, P-gp/MDR1 expression was downregulated across a large subset of studies. In contrast, functional P-gp inhibition without clear protein downregulation was observed with riccardin F, pakyonol, Euphorbia factor L1, ethyl lucidenates A, and DHH. This distinction is mechanistically important because it separates transcriptional or translational suppression of efflux proteins from direct functional interference with transporter activity. Agents in the latter group increased intracellular substrate accumulation or decreased efflux while leaving transporter abundance largely unchanged, suggesting transporter blockade or altered substrate handling rather than reduced protein expression.

**TABLE 9:** MDR transporter targets and pathway-level resistance mechanisms by study.

Mechanistic category	Specific marker/pathway	Direction or functional consequence	References
P-gp/MDR1 expression downregulation	P-gp/MDR1 decreased at mRNA, protein, promoter, or transcript level	Reduced efflux capacity and improved drug retention or sensitivity	(Li et al., 2012; Chen et al., 2016; Shu-Qi et al., 2011; Yi et al., 2021; Shen et al., 2025; Li et al., 2010; Min et al., 2017; Cai et al., 2019; Liu et al., 2021; Wang et al., 2016b; Wang et al., 2013; Wang et al., 2016c; Wang et al., 2016d; Han et al., 2018; Arken, 2019; Dechsupa et al., 2023)
P-gp functional inhibition without clear expression reduction	Increased substrate accumulation or reduced efflux without P-gp protein suppression	Direct transporter-function blockade	(Zhang et al., 2013; Ji et al., 2011; Li et al., 2019; Viriyaadhammaa et al., 2022)
MRP1/ABCC1 downregulation	MRP1 decreased or MRP1-mediated substrate accumulation improved	Reduced MRP1-associated efflux and restored anthracycline sensitivity	(Chen et al., 2016; Xu et al., 2014; Wang et al., 2022; Yi et al., 2018)
BCRP/ABCG2 modulation	BCRP-associated resistance phenotype reduced	Suppression of additional ABC transporter-mediated resistance	(Wang et al., 2022; Yi et al., 2021; Shen et al., 2025)
Increased intracellular anthracycline retention	ADR/DOX accumulation increased	Direct functional evidence of MDR reversal	(Li et al., 2012; Zhang et al., 2013; Chen et al., 2016; Ji et al., 2011; Shu-Qi et al., 2011; Xu et al., 2014; Wang et al., 2014a; Liu et al., 2021; Wang et al., 2013; Wang et al., 2016c; Wang et al., 2016d; Yi et al., 2018; Han et al., 2018; Arken, 2019; Dechsupa et al., 2023; Viriyaadhammaa et al., 2022)
Rh123, CFDA, VCR, bestatin, or THP transport modulation	Rh123/CFDA retention increased; THP/VCR/bestatin efflux reduced	Functional evidence of P-gp or MRP-related efflux inhibition	(Zhang et al., 2013; Chen et al., 2016; Ji et al., 2011; Shu-Qi et al., 2011; Xu et al., 2014; Shen et al., 2025; Min et al., 2017; Wang et al., 2016b; Wang et al., 2016c; Wang et al., 2016d; Han et al., 2018; Arken, 2019; Li et al., 2019; Dechsupa et al., 2023; Viriyaadhammaa et al., 2022)
NF-κB/IκB-α pathway inhibition	NF-κB activity, MDR1 promoter activity, or IκB-α activation decreased	Transcriptional suppression of MDR1/P-gp and apoptosis sensitization	(Yi et al., 2021; Wang et al., 2014a; Liu et al., 2021; Wang et al., 2013)
PI3K/Akt pathway inhibition	p-Akt or PI3K/Akt activity decreased	Downstream suppression of P-gp/MDR1, MRP1, survival signaling, or apoptosis resistance	(Chen et al., 2016; Xu et al., 2014; Wang et al., 2014a; Cai et al., 2019; Wang et al., 2016b; Wang et al., 2016c; Han et al., 2018; Arken, 2019)
PI3K/Akt/mTOR pathway inhibition	Akt/mTOR/S6RP/4EBP1 signaling reduced	Combined transporter modulation and apoptosis restoration	(Cai et al., 2019; Wang et al., 2016b)
Nrf2/ARE signaling inhibition	Nrf2 nuclear translocation, ARE binding, Nrf2 target genes, or Nrf2 protein level reduced	Downregulation of MRP1, P-gp, BCRP, and antioxidant defense-linked chemoresistance	(Xu et al., 2014; Shen et al., 2025)
JNK-associated MRP1 regulation	p-JNK/JNK linked to MRP1 suppression	MRP1-centered reversal mechanism	(Yi et al., 2018)
CXCL12/CXCR4-mediated survival signaling inhibition	CXCR4 and downstream PI3K/Akt/NF-κB signaling reduced	Reversal of microenvironment-related ADM resistance	(Wang et al., 2014a)
Autophagy/ERK-associated resistance-protein modulation	Autophagy markers and ERK activation linked to reduced resistance proteins	Resistance reversal through cytotoxic autophagy and transporter-marker reduction	(Wang et al., 2022; Liu et al., 2021)

MRP1 and BCRP also contributed to the MDR-reversal landscape. Table 9 indicates that MRP1/ABCC1 downregulation or functional modulation was reported in studies involving wogonin, timosaponin A-III, solanine, and S-20, while BCRP/ABCG2 modulation was implicated in studies involving S-20, parthenolide, and TMI. These findings broaden the mechanistic scope beyond P-gp-centered reversal and suggest that selected natural products may target multidrug resistance through multiple ABC transporters. This is particularly relevant for resistant phenotypes in which P-gp alone may not fully explain drug resistance, or where antioxidant defense pathways and stem-like resistant subpopulations contribute to reduced chemosensitivity.

Functional substrate-accumulation and efflux assays provided important confirmatory evidence for transporter modulation. Table 10 shows that many studies measured intracellular accumulation of ADM, ADR, DOX, Rh123, CFDA, THP, VCR, or bestatin. Increased anthracycline accumulation was reported across several K562/A02, K562/ADM, and K562/ADR studies, indicating restoration of intracellular drug exposure after treatment with natural product-derived interventions. In addition, Rh123, CFDA, THP, VCR, and bestatin transport assays supported functional modulation of P-gp- or MRP-related efflux. These assays strengthen the interpretation that the observed IC50 reductions were not merely nonspecific cytotoxic effects, but were often linked to restoration of intracellular drug retention.

**TABLE 10:** Functional substrate-accumulation and efflux assays supporting MDR reversal.

Ref.	Intervention	Functional substrate assay	Reported functional effect	Transporter interpretation
(Li et al., 2012)	Triptolide	ADM accumulation	Intracellular ADM accumulation increased	Consistent with reduced P-gp/MDR1-mediated efflux
(Ji et al., 2011)	Riccardin F; pakyonol	ADR accumulation; Rh123 accumulation	ADR accumulation increased 4.3-fold and 5.7-fold; Rh123 accumulation increased by 90.3% and 151.7%	Strong functional P-gp inhibition
(Shu-Qi et al., 2011)	Archangelicin	Dox accumulation; Rh123 accumulation/efflux	Dox/Rh123 accumulation increased and efflux decreased	Functional P-gp inhibition plus expression downregulation
(Zhang et al., 2013)	Euphorbia factor L1	DOX accumulation; Rh123 accumulation/efflux	DOX/Rh123 accumulation increased and Rh123 efflux inhibited	Functional ABCB1/P-gp blockade without ABCB1 protein downregulation
(Wang et al., 2014a)	Oroxylin A	ADM sensitization with pathway controls	ADM sensitivity improved alongside CXCR4 pathway inhibition	Resistance reversal linked mainly to survival signaling rather than direct efflux assay only
(Xu et al., 2014)	Wogonin	ADR accumulation; CFDA accumulation	Intracellular ADR/CFDA accumulation increased	MRP1 functional inhibition/downregulation through Nrf2/ARE suppression
(Chen et al., 2016)	Timosaponin A-III	ADM, Rho-123, and CFDA accumulation	Accumulation of transporter substrates increased	Dual P-gp/MRP1 functional reversal
(Wang et al., 2013)	Dioscin	ADR accumulation	Intracellular ADR accumulation recovered	MDR1 suppression restored intracellular drug retention
(Wang et al., 2016c)	Osthole	Rh123 uptake/efflux; DOX accumulation	Intracellular Rh123 and DOX accumulation increased	P-gp-mediated efflux inhibition through PI3K/Akt suppression
(Wang et al., 2016d)	Resveratrol	Rh123 retention; ADM accumulation	Rh123 retention and ADM accumulation increased	P-gp inhibition/downregulation despite modest ADM RF
(Min et al., 2017)	Emodin	Rh123 uptake; emodin uptake under P-gp-inhibited conditions	Rh123 uptake increased; kinetic data supported P-gp interaction	Competitive inhibition and possible substrate behavior at P-gp binding sites
(Yi et al., 2018)	Solanine	ADR accumulation	Intracellular ADR accumulation increased	MRP1 downregulation increased ADR retention
(Han et al., 2018)	Ailanthone	DOX accumulation; Rh123 efflux; ATPase assay	DOX retention increased; Rh123 efflux decreased; P-gp ATPase activity modulated	Potent P-gp functional inhibition and efflux reversal
(Arken, 2019)	Schizandrol A	DOX accumulation; Rh123 efflux	DOX accumulation increased and Rh123 efflux decreased	P-gp function inhibition and p-Akt/P-gp downregulation
(Liu et al., 2021)	Puerarin	ADR accumulation	ADR accumulation increased	MDR1 downregulation restored intracellular anthracycline exposure
(Wang et al., 2016b)	Resveratrol	Bestatin accumulation	Intracellular bestatin concentration increased	P-gp-mediated non-anthracycline substrate retention improved
(Li et al., 2019)	Ethyl lucidenates A	Rh123 accumulation; VCR resistance assay	Rh123 accumulation increased and VCR RF reached 7.59	Functional P-gp inhibition without expression reduction
(Dechsupa et al., 2023)	Pentagalloyl glucose	THP efflux kinetics; intracellular drug accumulation	P-gp-mediated efflux inhibited and intracellular drug accumulation increased	P-gp expression and transport function both suppressed
(Viriyaadhammaa et al., 2022)	DHH	THP efflux kinetics; DOX/THP accumulation	Active efflux reduced and drug accumulation increased	Functional P-gp blockade without P-gp expression reduction
(Shen et al., 2025)	TMI	Rh123 accumulation	Rh123 accumulation increased	Reduced drug-efflux activity consistent with Nrf2-mediated P-gp/BCRP downregulation

Pathway-level regulation was also prominent. Table 9 shows that NF-κB/IκB-α signaling was linked to MDR1/P-gp transcriptional suppression and apoptosis sensitization, particularly in studies involving oroxylin A, dioscin, puerarin, and parthenolide. PI3K/Akt signaling was another recurrent pathway, implicated in transporter regulation, survival signaling, and apoptosis resistance in studies involving oroxylin A, wogonin, timosaponin A-III, osthole, ailanthone, schizandrol A, costunolide, and resveratrol. Nrf2/ARE inhibition was associated with reduced MRP1, P-gp, BCRP, and antioxidant-defense signaling, while JNK, ERK/autophagy, and CXCL12/CXCR4 mechanisms were more specific to intervention contexts.

### 3.5 Cell-death and signaling pathways associated with MDR reversal

Cell death restoration and modulation of signaling pathways were recurrent mechanistic features of MDR reversal across the included resistant K562 models. As summarized in Table 11, the reviewed studies linked natural product-mediated chemosensitization to apoptosis induction, transporter-associated survival signaling, autophagy-related mechanisms, cell-cycle perturbation, and regulation of upstream pathways such as NF-κB/IκB-α, PI3K/Akt, PI3K/Akt/mTOR, Nrf2/ARE, JNK, ERK/autophagy, and CXCL12/CXCR4. These findings indicate that MDR reversal was not limited to direct inhibition of drug-efflux transporters, but often involved broader reprogramming of pro-survival and cell-death regulatory networks.

**TABLE 11:** Study-level cell-death and signaling outcomes associated with MDR reversal.

Ref.	Intervention	Signaling pathway or regulatory axis	Direction of effect	Interpretation for MDR reversal
(Li et al., 2010)	Haishengsu	Apoptosis-associated caspase-3/Bcl-2 balance; P-gp reduction	Apoptosis increased dose- and time-dependently; caspase-3 increased; Bcl-2 and P-gp decreased	Haishengsu suggests that MDR reversal may involve simultaneous reduction of efflux phenotype and restoration of apoptosis susceptibility
(Li et al., 2012)	Triptolide	MDR1 promoter suppression; P-gp reduction; possible NF-κB-related transcriptional modulation	ADM-induced apoptosis increased after triptolide co-treatment; P-gp/MDR1 promoter activity decreased	Triptolide links transcriptional MDR1 suppression with enhanced ADM-induced apoptosis
(Ji et al., 2011)	Riccardin F; pakyonol	P-gp transport inhibition	Cell-death signaling not a main endpoint	Primary evidence supports functional efflux blockade rather than apoptosis-pathway modulation
(Shu-Qi et al., 2011)	Archangelicin	P-gp functional inhibition plus MDR1/P-gp downregulation	Cell-death signaling not a main endpoint	Mechanistic value is mainly transporter-centered rather than apoptosis-centered
(Wang et al., 2011)	Coumarins from *Cicuta virosa*	P-gp-mediated MDR inferred	Cell-death signaling not assessed in depth	Metabolite-screening evidence for MDR reversal without detailed cell-death mechanism
(Wang et al., 2016a)	*Cicuta virosa* metabolites 1–9	P-gp-mediated MDR inferred	Cell-death signaling not assessed in depth	Metabolite-screening evidence for DOX reversal with limited pathway characterization
(Zhang et al., 2013)	Euphorbia factor L1	Mitochondrial apoptosis pathway; caspase-3; PARP; cytochrome c; ABCB1 functional inhibition	VCR-induced apoptosis increased after EFL1 co-treatment; caspase-3, cleaved PARP, and cytochrome c release increased	EFL1 demonstrates that functional ABCB1 inhibition can restore apoptotic response without downregulating ABCB1 expression
(Wang et al., 2014a)	Oroxylin A	CXCL12/CXCR4 → PI3K/Akt → NF-κB; survivin; p-BAD	Oroxylin A enhanced ADM-induced apoptosis and reduced CXCR4, p-Akt, NF-κB activation, survivin, and p-BAD	MDR reversal was associated with suppression of microenvironment-related survival signaling and apoptosis restoration
(Xu et al., 2014)	Wogonin	Nrf2/ARE/MRP1 axis; PI3K/Akt/DNA-PKcs involvement	Wogonin enhanced ADR-induced apoptosis, reduced Ki67, suppressed Nrf2/ARE activity, and downregulated MRP1	Wogonin connects apoptosis restoration to inhibition of antioxidant-defense and MRP1-mediated resistance signaling
(Chen et al., 2016)	Timosaponin A-III	PI3K/Akt suppression; P-gp/MRP1 downregulation	TAIII plus ADM sensitized cells and induced apoptosis dose-dependently; p-Akt, P-gp, and MRP1 decreased	TAIII supports a dual-transporter and survival-pathway model of MDR reversal
(Wang et al., 2013)	Dioscin	NF-κB/IκB-α → MDR1 axis	NF-κB activity and MDR1 promoter activity decreased; intracellular ADR accumulation increased	Dioscin restored ADR activity mainly through transcriptional suppression of MDR1 rather than a primarily apoptosis-focused endpoint
(Wang et al., 2016c)	Osthole	PI3K/Akt → P-gp/MDR1	P-gp/MDR1 and p-Akt decreased; DOX and Rh123 accumulation increased	Osthole links P-gp-mediated reversal to PI3K/Akt pathway inhibition
(Wang et al., 2016d)	Resveratrol	P-gp/MDR1 downregulation	ADM accumulation and Rh123 retention increased; P-gp/MDR1 decreased	Resveratrol provides transporter-related sensitization evidence with modest ADM IC50 reduction
(Min et al., 2017)	Emodin	P-gp competitive inhibition and downregulation	Apoptosis increased when ADR was combined with emodin; P-gp expression and efflux activity decreased	Emodin demonstrates that P-gp inhibition/downregulation can enhance ADR-triggered apoptosis
(Yi et al., 2018)	Solanine	JNK → MRP1 axis	MRP1 decreased and ADR accumulation increased; JNK signaling implicated	Solanine supports an MRP1/JNK-centered mechanism rather than a primary apoptosis mechanism
(Han et al., 2018)	Ailanthone	PI3K/Akt → MDR1/P-gp; P-gp ATPase/efflux	P-gp function and MDR1/P-gp expression decreased	Ailanthone mainly acts as a potent P-gp reversal agent; cell-death signaling was secondary to transporter inhibition
(Arken, 2019)	Schizandrol A	PI3K/Akt → P-gp; Rh123 efflux blockade	P-gp and p-Akt decreased; DOX accumulation increased	Schizandrol A links P-gp functional inhibition to PI3K/Akt-associated regulation
(Cai et al., 2019)	Costunolide	PI3K/Akt/mTOR/S6RP/4EBP1 pathway; P-gp downregulation	Combination treatment markedly increased apoptosis; caspase-3 activation and PARP cleavage increased; PI3K/Akt/mTOR signaling decreased	Costunolide supports survival-pathway inhibition and apoptosis restoration as metabolites of DOX sensitization
(Liu et al., 2021)	Puerarin	NF-κB/IκB-α → MDR1; Akt/JNK; LC3-II; Beclin-1	MDR1 decreased; NF-κB/IκB-α activation suppressed; autophagosomes, LC3-II, and Beclin-1 increased; Akt/JNK phosphorylation decreased	Puerarin links MDR reversal to both NF-κB-mediated MDR1 suppression and autophagy-associated cell-death signaling
(Wang et al., 2016b)	Resveratrol + bestatin	PI3K/Akt/mTOR → P-gp/MDR1	Resveratrol enhanced bestatin-induced caspase-3/8 activation, increased bestatin accumulation, and reduced P-gp/MDR1 and Akt/mTOR signaling	Mechanistically relevant to K562/ADR MDR, although the substrate is bestatin rather than ADM/DOX
(Ma et al., 2017)	*Woodwardia unigemmata* flavonoids	MDR reversal screening; BSA-binding/pharmacokinetic support	Cell-death signaling not characterized in depth	Primarily useful for DOX reversal screening and metabolite-distribution discussion
(Wang et al., 2014b)	*Ustilago maydis* metabolites	Metabolite-screening MDR assay	Cell-death signaling not characterized in depth	Modest DOX reversal evidence without detailed cell-death or signaling analysis
(Li et al., 2019)	Ethyl lucidenates A	P-gp functional inhibition; P-gp expression unchanged	VCR plus ethyl lucidenates A induced G2/M arrest; Rh123 accumulation increased	Ethyl lucidenates A suggests that functional P-gp inhibition can be coupled with cell-cycle disruption under VCR exposure
(Dechsupa et al., 2023)	Pentagalloyl glucose	P-gp expression/function; docking to substrate and ATP-binding sites	PGG selectively induced cytotoxicity in K562/ADR cells, enhanced DOX sensitivity, and inhibited P-gp expression/function	PGG integrates apoptosis-related cytotoxicity with direct and expression-level P-gp inhibition
(Wang et al., 2022)	S-20	Autophagy → ERK activation → resistance-protein reduction	S-20 induced apoptosis and autophagic cell death; ERK activation was triggered by autophagy and contributed to drug-resistance protein downregulation	S-20 provides a non-classical anti-MDR mechanism based on cytotoxic autophagy and ERK-mediated reduction of resistance proteins
(Yi et al., 2021)	Parthenolide	NF-κB suppression → P-gp downregulation; ROS and mitochondrial-dependent apoptosis	PTL enhanced DOX-induced apoptosis and suppressed aberrant NF-κB/P-gp signaling in K562/ADM and drug-resistant LSC contexts	PTL links MDR reversal to leukemia stem-cell targeting and NF-κB/P-gp suppression
(Viriyaadhammaa et al., 2022)	DHH	P-gp functional inhibition; THP efflux kinetic suppression; P-gp expression unchanged	DHH increased intracellular drug accumulation and enhanced apoptosis without reducing P-gp expression	DHH supports a functional P-gp blockade model leading to restored drug-induced apoptosis
(Shen et al., 2025)	TMI	Direct Nrf2 targeting/degradation → P-gp/BCRP downregulation	TMI induced G2/M arrest and caspase-dependent apoptosis; P-gp/BCRP decreased through Nrf2 targeting; ROS, MAPK, and PI3K/Akt/mTOR were not essential for resistance-overcoming effect	TMI provides a Nrf2-centered model linking transporter downregulation, cell-cycle arrest, and apoptosis in K562/ADR cells

Table 11 shows that several interventions enhanced apoptosis or restored drug-induced apoptotic responsiveness in resistant K562 cells. Haishengsu increased apoptosis while upregulating caspase-3 and reducing Bcl-2 and P-gp, suggesting coordinated modulation of apoptotic and efflux-associated markers. Triptolide enhanced ADM-induced apoptosis in K562/A02 cells while suppressing P-gp and MDR1 promoter activity. Similarly, emodin, costunolide, parthenolide, DHH, and TMI were associated with increased apoptosis or apoptosis-related signaling in resistant cells. These findings support a mechanistic interpretation in which MDR reversal is coupled to restoration of drug-induced cell death rather than being explained solely by changes in IC50 values.

The quantitative apoptosis data in Table 12 further support this interpretation. Haishengsu induced a dose-dependent increase in apoptosis at 36 h, rising from a low baseline in untreated K562/ADM cells to markedly higher levels at the highest tested concentration. Triptolide increased ADM-induced apoptosis in K562/A02 cells, while EFL1 enhanced VCR-induced apoptosis in K562/ADR cells and increased downstream apoptotic markers, including caspase-3 and cleaved PARP. Emodin also increased ADR-induced apoptosis in a dose-related manner, and costunolide produced a strong apoptotic response when combined with DOX. Although the apoptosis endpoints were heterogeneous and not uniformly suitable for pooled quantitative synthesis, they provide consistent biological support for cell-death restoration as a secondary mechanism of MDR reversal.

**TABLE 12:** Quantitative apoptosis and cell-death outcomes reported in the included studies.

Ref.	Intervention/combination	Quantitative cell-death outcome	Direction of effect	Extraction relevance
(Li et al., 2010)	Haishengsu 10, 20, 40 μg/mL	At 36 h, apoptosis increased to 5.54% ± 2.54%, 9.35% ± 3.21%, and 35.45% ± 3.21%, respectively, versus 2.31% ± 1.24% in control cells	Dose-dependent apoptosis induction	Suitable for apoptosis-focused synthesis rather than ADM/DOX IC50 pooling
(Li et al., 2012)	ADM + triptolide	ADM-induced apoptosis increased from 4.3% with ADM alone to 18.5% with ADM + triptolide	Enhanced apoptosis after sensitization	Graph-based or limited quantitative apoptosis synthesis
(Zhang et al., 2013)	VCR + EFL1	VCR-induced apoptosis increased from 15.5% ± 1.2% with VCR alone to 36.2% ± 3.6% with VCR + EFL1; caspase-3/GAPDH increased to 49.90% ± 3.56%; cleaved PARP/GAPDH increased to 74.82% ± 6.62%	Strong apoptosis sensitization	Useful for non-DOX apoptosis mechanism; VCR substrate requires separate interpretation
(Min et al., 2017)	ADR + emodin 10 or 20 µM	ADR-induced apoptosis increased from 11% with ADR alone to 29.3% and 38.3% with ADR plus 10 and 20 µM emodin, respectively	Dose-related apoptosis enhancement	Suitable for apoptosis-restoration narrative and possible SMD extraction if denominator/variance are available
(Cai et al., 2019)	Costunolide 15 µM + DOX 10 µM	Apoptosis reached 37.56% ± 4.18% with combination treatment versus 2.11% ± 0.61% control, 18.22% ± 3.96% costunolide alone, and 9.09% ± 3.88% DOX alone	Synergistic apoptosis enhancement	Suitable for apoptosis outcome synthesis; not a simple RF endpoint
(Liu et al., 2021)	Puerarin + ADR	Autophagy induction, cell-cycle arrest, and apoptosis enhancement reported; LC3-II and Beclin-1 increased	Autophagy-associated sensitization and apoptosis/cell-cycle modulation	Best used for mechanistic synthesis; specific numeric extraction may require figure digitization
(Wang et al., 2016b)	Resveratrol + bestatin	Caspase-3 and caspase-8 activation increased after combination treatment	Enhanced bestatin-induced apoptosis	Relevant to MDR apoptosis mechanism but not ADM/DOX endpoint
(Dechsupa et al., 2023)	PGG + DOX	Apoptosis assessed by flow cytometry; PGG selectively induced cytotoxicity and enhanced DOX sensitivity	Apoptosis/cytotoxic sensitization	Mechanistic and potential apoptosis synthesis; numeric extraction may require figure/table digitization
(Wang et al., 2022)	S-20	Caspase-dependent apoptosis and autophagic cell death reported	Direct cytotoxicity against resistant cells and anti-MDR activity	Mechanistic synthesis; not conventional ADM/DOX co-treatment RF
(Yi et al., 2021)	PTL + DOX	PTL enhanced DOX-induced apoptosis and eliminated drug-resistant LSCs	Apoptosis enhancement and LSC targeting	Mechanistic synthesis; K562/ADM data should remain separate from LSC findings
(Viriyaadhammaa et al., 2022)	DHH + DOX/THP	Apoptosis enhanced alongside increased intracellular drug accumulation	Drug-accumulation-linked apoptosis	Mechanistic synthesis; exact quantitative pooling requires figure extraction
(Shen et al., 2025)	TMI	G2/M arrest and caspase-dependent apoptosis reported	Cell-cycle arrest and apoptosis induction	Mechanistic synthesis linked to Nrf2/P-gp/BCRP regulation

Figure 5A provides a pathway-level visualization of the intervention–signaling associations identified across the included studies. The heatmap demonstrates that multiple interventions converged on a limited set of recurring regulatory axes, particularly PI3K/Akt signaling, mitochondrial/caspase-PARP apoptosis, and NF-κB/IκB-α signaling. These recurring associations were reported across several distinct metabolite studies and do not establish a shared pharmacological mechanism across metabolites and resistant sublines. The figure also shows more selective pathway associations, including CXCL12/CXCR4 modulation by oroxylin A, ERK/autophagy signaling by S-20, and Nrf2/ARE regulation by wogonin and TMI.

**FIGURE 5:**
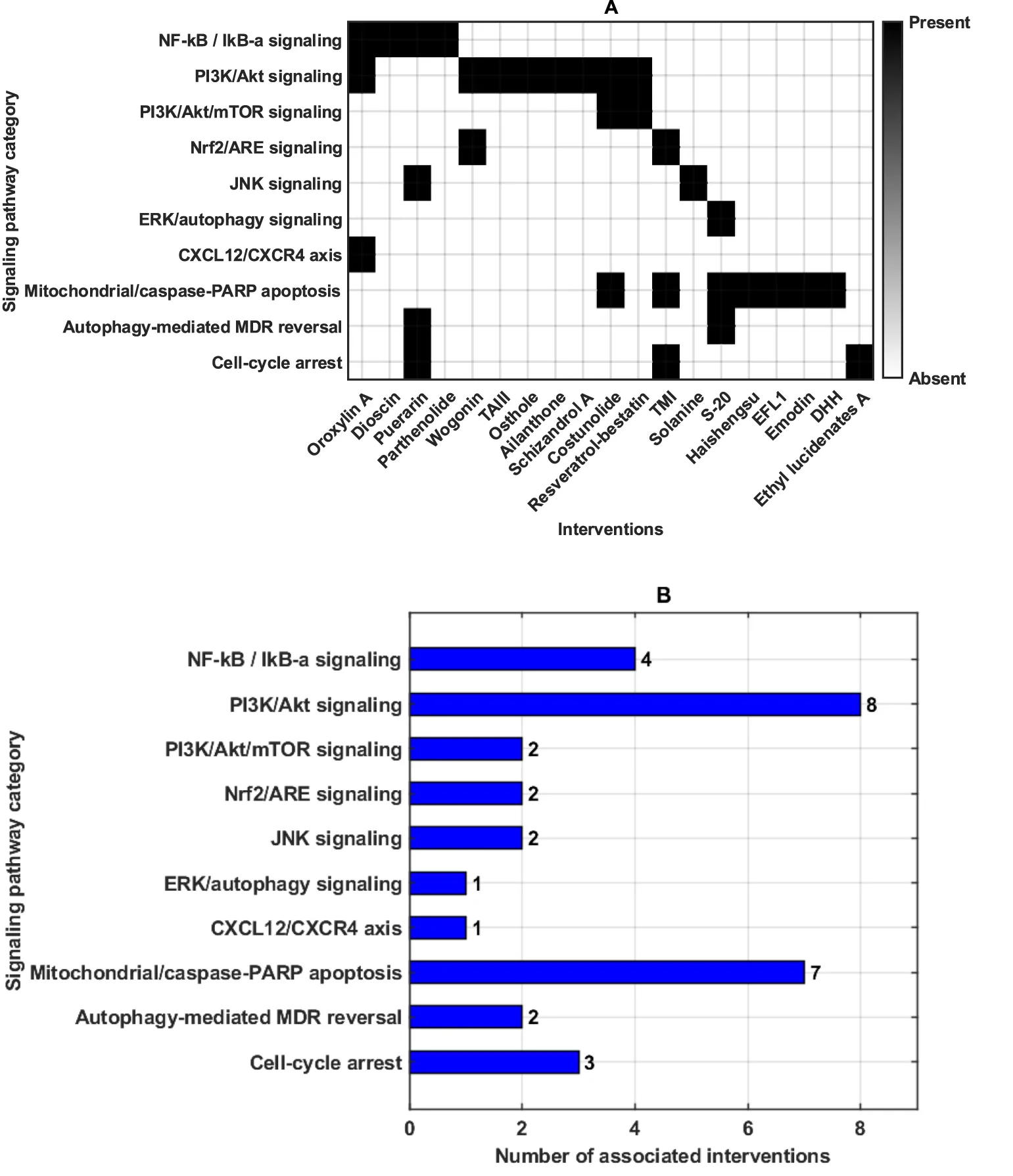
Signaling pathway categories implicated in MDR reversal and cell-death restoration. **(A)** Intervention–pathway association heatmap. **(B)** Frequency of intervention involvement across pathway categories.

The frequency distribution in Figure 5B reinforces the predominance of PI3K/Akt-related signaling, which was associated with the largest number of interventions. This pathway was observed in studies involving oroxylin A, wogonin, timosaponin A-III, osthole, ailanthone, schizandrol A, costunolide, and resveratrol-bestatin, indicating a recurrent association between PI3K/Akt suppression, transporter regulation, and improved drug sensitivity. Mitochondrial/caspase-PARP apoptosis was also frequently represented, reflecting the importance of apoptotic execution after restoration of intracellular drug exposure or suppression of survival signaling. NF-κB/IκB-α signaling formed another important pathway cluster, particularly in studies linking MDR1/P-gp transcriptional suppression to chemosensitization.

Autophagy and cell-cycle regulation were less frequent but mechanistically important. Table 11 and Figure 5 identify puerarin and S-20 as interventions linked to autophagy-associated MDR reversal, with S-20 providing evidence for autophagic cell death and ERK activation as part of resistance-protein reduction. Cell-cycle arrest was reported in association with ethyl lucidenates A, puerarin, and TMI, suggesting that some natural product-derived interventions may reduce the viability of resistant cells or enhance drug response by disrupting cell-cycle pathways in addition to transporter modulation. These mechanisms appear more selective than PI3K/Akt or caspase-mediated apoptosis, but they expand the mechanistic scope of MDR reversal beyond classical efflux inhibition.

### 3.6 Meta-analytic findings, heterogeneity, and sensitivity analyses

Nineteen study-level effects were included in the primary random-effects meta-analysis using log-transformed reversal fold [log (RF)] as the effect size. Table 7 presents the extracted IC50 values, standard deviations, RF estimates, log (RF), standard errors, variances, and random-effects weights.

The overall pooled RF was 6.31, with a 95% CI of 4.77–8.36, summarizing a common chemosensitization endpoint across the restricted quantitative dataset, while effect magnitude varied substantially across resistant K562 models. These pooled estimates are reported in Table 13 and visualized in Figure 6. Heterogeneity was high, with Q = 772.34, p < 0.001, I^2^ = 97.7%, and τ^2^ = 0.379. The 95% prediction interval ranged from 1.66 to 23.96. Subgroup analysis in Table 13 showed pooled RF values of 5.71 for K562/A02, 4.90 for K562/ADM, and 8.91 for K562/ADR, with a significant subgroup difference (Q = 122.95, p < 0.001).

**TABLE 13:** Overall and K562-subline subgroup random-effects meta-analysis.

Analysis group	Studies/effects	Pooled log (RF)	SE	Pooled RF	95% CI	Cochran Q	Q p-value	I^2^ (%)	τ^2^	95% prediction interval
Overall	19	1.843	0.143	6.31	4.77–8.36	772.34	<0.001	97.7	0.379	1.66–23.96
K562/A02	8	1.743	0.207	5.71	3.81–8.56	281.70	<0.001	97.5	0.334	1.27–25.64
K562/ADM	5	1.590	0.250	4.90	3.01–8.00	135.35	<0.001	97.0	0.303	0.72–33.60
K562/ADR	6	2.187	0.275	8.91	5.20–15.27	232.34	<0.001	97.8	0.445	1.20–66.06
Subgroup difference	3 groups	—	—	—	—	122.95	<0.001	—	—	—

**FIGURE 6:**
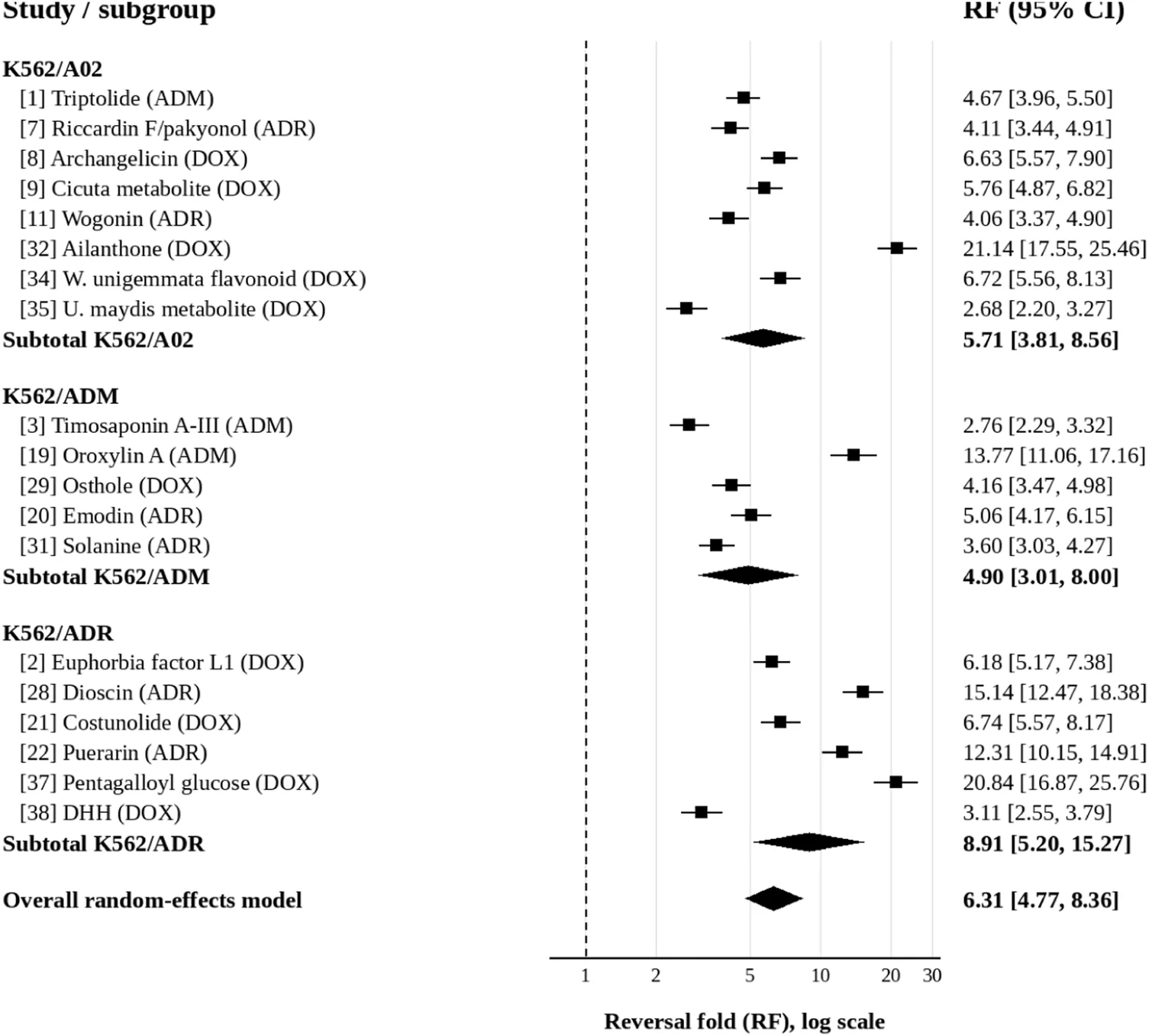
Forest plot of random-effects reversal-fold effects across resistant K562 models.

Table 14 shows that the pooled chemosensitization signal remained positive across all sensitivity scenarios. Excluding high-effect studies reduced the pooled RF to 4.51, whereas other sensitivity scenarios produced pooled RF values ranging from 6.09 to 6.97. Table 15 shows that leave-one-out pooled RF estimates ranged from 5.90 to 6.62, indicating that no single study fully determined the overall result.

**TABLE 14:** Sensitivity analyses of the primary pooled RF estimate.

Sensitivity scenario	Excluded/modified effects	Effects	Pooled RF	95% CI	I^2^ (%)	τ^2^	Change from main (%)
Main random-effects model	None	19	6.31	4.77–8.36	97.7	0.379	0.0
Excluding high-effect studies	(Wang et al., 2014a; Liu et al., 2021; Wang et al., 2013; Han et al., 2018; Dechsupa et al., 2023)	14	4.51	3.57–5.69	92.8	0.113	−28.5
Excluding graph-derived/less precise estimates	(Wang et al., 2014b; Viriyaadhammaa et al., 2022)	17	6.92	5.17–9.25	97.5	0.382	+9.6
Excluding high-risk screening studies	(Wang et al., 2011; Ma et al., 2017; Wang et al., 2014b)	16	6.67	4.88–9.13	97.8	0.423	+5.7
Excluding modest-effect studies	(Chen et al., 2016; Wang et al., 2014b)	17	6.97	5.30–9.16	96.6	0.291	+10.4
Inflated variance scenario	All SE values increased by 25%	19	6.31	4.58–8.67	96.3	0.379	−0.1
Fixed-effect sensitivity model	None	19	6.09	5.80–6.40	97.7	0.000	−3.5

**TABLE 15:** Leave-one-out influence analysis.

Omitted ref.	Omitted intervention	Model	Pooled RF after omission	95% CI	I^2^ (%)	Change from main (%)
(Han et al., 2018)	Ailanthone	K562/A02	5.90	4.55–7.65	97.1	−6.5
(Dechsupa et al., 2023)	Pentagalloyl glucose	K562/ADR	5.91	4.52–7.73	97.4	−6.4
(Wang et al., 2013)	Dioscin	K562/ADR	6.02	4.56–7.94	97.5	−4.7
(Wang et al., 2014a)	Oroxylin A	K562/ADM	6.05	4.56–8.03	97.6	−4.2
(Liu et al., 2021)	Puerarin	K562/ADR	6.08	4.58–8.09	97.6	−3.6
(Cai et al., 2019)	Costunolide	K562/ADR	6.29	4.68–8.46	97.8	−0.4
(Ma et al., 2017)	W. unigemmata flavonoid	K562/A02	6.29	4.68–8.46	97.8	−0.3
(Shu-Qi et al., 2011)	Archangelicin	K562/A02	6.30	4.68–8.48	97.8	−0.3
(Zhang et al., 2013)	Euphorbia factor L1	K562/ADR	6.32	4.70–8.51	97.8	+0.1
(Wang et al., 2011)	Cicuta metabolite	K562/A02	6.35	4.71–8.55	97.8	+0.5
(Min et al., 2017)	Emodin	K562/ADM	6.39	4.76–8.59	97.8	+1.2
(Li et al., 2012)	Triptolide	K562/A02	6.42	4.78–8.64	97.8	+1.7
(Wang et al., 2016c)	Osthole	K562/ADM	6.46	4.82–8.67	97.7	+2.4
(Ji et al., 2011)	Riccardin F/pakyonol	K562/A02	6.47	4.82–8.67	97.7	+2.4
(Xu et al., 2014)	Wogonin	K562/A02	6.47	4.83–8.67	97.7	+2.5
(Yi et al., 2018)	Solanine	K562/ADM	6.52	4.88–8.71	97.7	+3.2
(Viriyaadhammaa et al., 2022)	DHH	K562/ADR	6.57	4.93–8.74	97.7	+4.0
(Chen et al., 2016)	Timosaponin A-III	K562/ADM	6.61	4.99–8.76	97.6	+4.7
(Wang et al., 2014b)	U. maydis metabolite	K562/A02	6.62	4.99–8.78	97.6	+4.9

Figure 7 shows mild right-sided funnel-plot asymmetry. Egger’s test did not reach statistical significance, with an intercept of 36.98, SE of 19.86, t = 1.86, and p = 0.080. Trim-and-fill analysis imputed data from three studies and yielded an adjusted pooled RF of 5.48 (95% CI, 3.87–7.76), indicating that the overall effect remained positive after adjustment.

**FIGURE 7:**
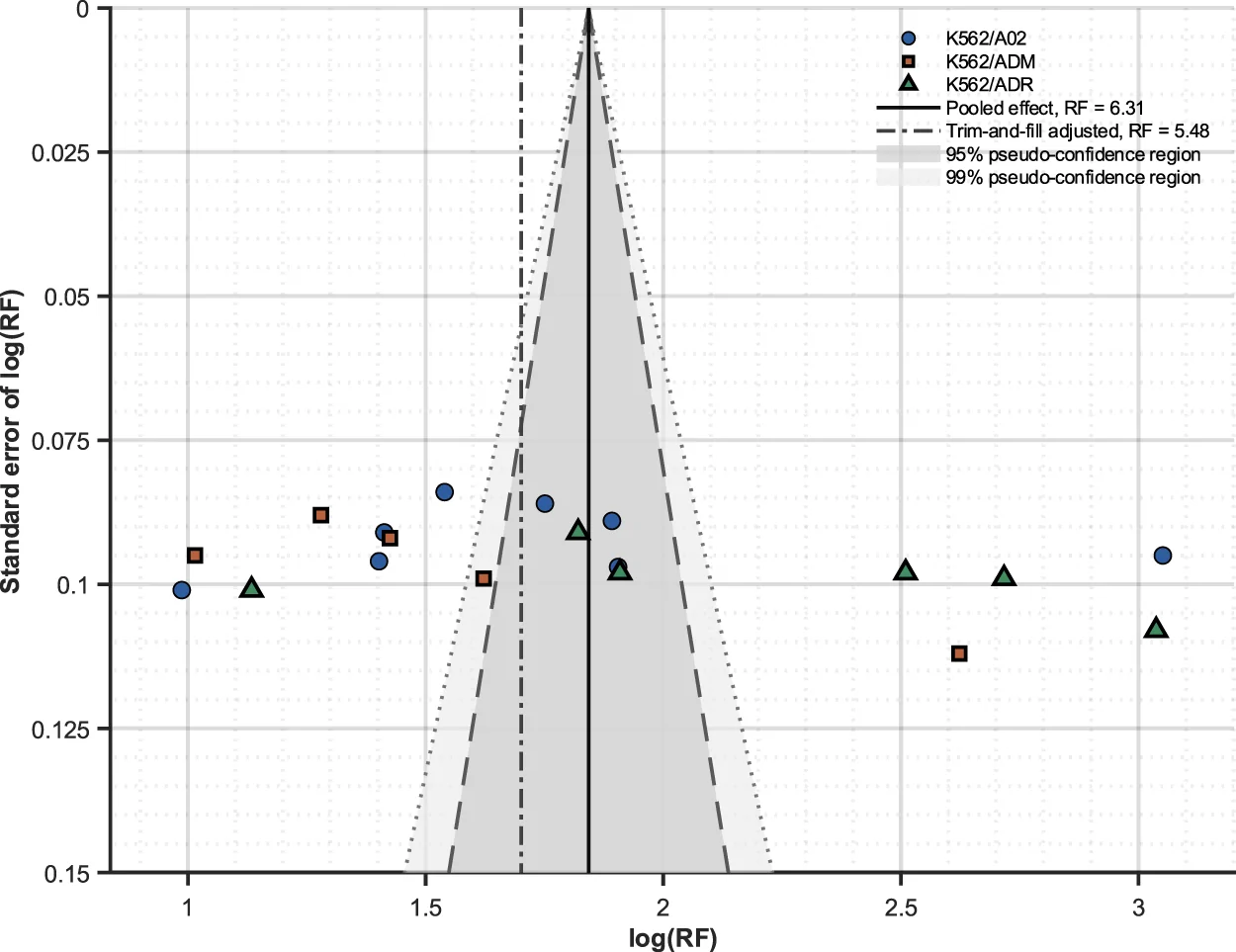
Funnel plot for small-study effects in the primary random-effects meta-analysis.

### 3.7 Quality assessment and risk of bias

The methodological quality assessment indicated that the included evidence base was generally suitable for structured qualitative synthesis and RF-based quantitative analysis, although several recurring concerns required careful analytical handling. As summarized in Table 16, the most frequent issue was the presence of multiple-arm or shared-comparator designs, which affected 11 studies and required either preferred-arm selection or conservative analytical treatment to avoid unit-of-analysis distortion. Additional important concerns included dose-selection uncertainty, the presence of studies without a strict ADM/DOX RF endpoint, graph-based or non-tabulated quantitative outcomes, and a subset of high-effect estimates that could exert disproportionate influence on the quantitative synthesis.

**TABLE 16:** Frequency of major methodological concerns affecting synthesis.

Methodological concern	Definition used in this review	No. of affected studies	References	Implication for analysis
No direct strict ADM/DOX RF endpoint	Study lacks a directly comparable ADM/DOX IC50/RF endpoint or uses non-standard resistance-overcoming outcome	5	(Wang et al., 2022; Shen et al., 2025; Li et al., 2010; Wang et al., 2016b; Li et al., 2019)	Exclude from strict primary ADM/DOX RF pool; retain for mechanistic synthesis
Graph-based or non-tabulated quantitative sensitization	Key quantitative outcome requires figure digitization or is not tabulated as RF	4	(Li et al., 2012; Yi et al., 2021; Cai et al., 2019; Arken, 2019)	Use only after calibrated extraction; test by sensitivity analysis
Multiple-arm/shared-comparator issue	Several doses or metabolites share one resistant-cell comparator	11	(Zhang et al., 2013; Chen et al., 2016; Ji et al., 2011; Wang et al., 2011; Wang et al., 2016a; Xu et al., 2014; Wang et al., 2014a; Wang et al., 2016c; Wang et al., 2016d; Ma et al., 2017; Wang et al., 2014b)	Use preferred-arm extraction or comparator splitting
High-effect estimate	Preferred RF or reversal index ≥10	5	(Wang et al., 2014a; Liu et al., 2021; Wang et al., 2013; Han et al., 2018; Dechsupa et al., 2023)	Perform outlier and leave-one-out sensitivity analyses
Potential metabolite/dataset overlap	Same or closely related metabolite-condition may appear in more than one report	2	(Shu-Qi et al., 2011; Wang et al., 2011)	Avoid double counting archangelicin by selecting (Shu-Qi et al., 2011) for archangelicin and metabolite 7 from (Wang et al., 2011)
Non-anthracycline primary substrate	Main reversal substrate is VCR or bestatin rather than ADM/DOX	2	(Wang et al., 2016b; Li et al., 2019)	Keep outside strict ADM/DOX quantitative pool
Mechanistic inference without detailed transporter assays	RF/IC50 screening without strong transporter/pathway validation	4	(Wang et al., 2011; Wang et al., 2016a; Ma et al., 2017; Wang et al., 2014b)	Interpret as screening evidence; use mechanistic claims cautiously
Subline/model complexity	Study includes LSCs, *in vivo* metabolites, or non-standard cell-population context alongside K562 data	3	(Yi et al., 2021; Wang et al., 2014a; Wang et al., 2016d)	Extract only *in vitro* resistant K562 data for primary synthesis; discuss additional models separately
Dose-selection uncertainty	Higher dose may produce stronger RF but cytotoxicity or concentration selection may affect interpretation	6	(Wang et al., 2014a; Min et al., 2017; Liu et al., 2021; Wang et al., 2013; Han et al., 2018; Dechsupa et al., 2023)	Conduct conservative dose or high-effect sensitivity analyses

The study-level concern profile is visualized in Figure 8A, which demonstrates that the methodological limitations were not uniformly distributed across the evidence base. Instead, specific studies tended to cluster around particular concern domains. For example, non-strict primary endpoints were concentrated in a small subset of studies, which were retained primarily for mechanistic interpretation rather than for strict RF pooling. Likewise, graph-based or non-tabulated outcomes were limited to a defined group of studies, supporting the decision to treat these data cautiously and, where necessary, reserve them for secondary or narrative synthesis. The figure also shows that high-effect influence was restricted to a relatively small number of studies, whereas multiple-arm or dose-related concerns were more widely distributed. This pattern indicates that the principal analytical challenge in the review was not widespread poor study quality, but rather heterogeneity in outcome structure, reporting style, and experimental design.

**FIGURE 8:**
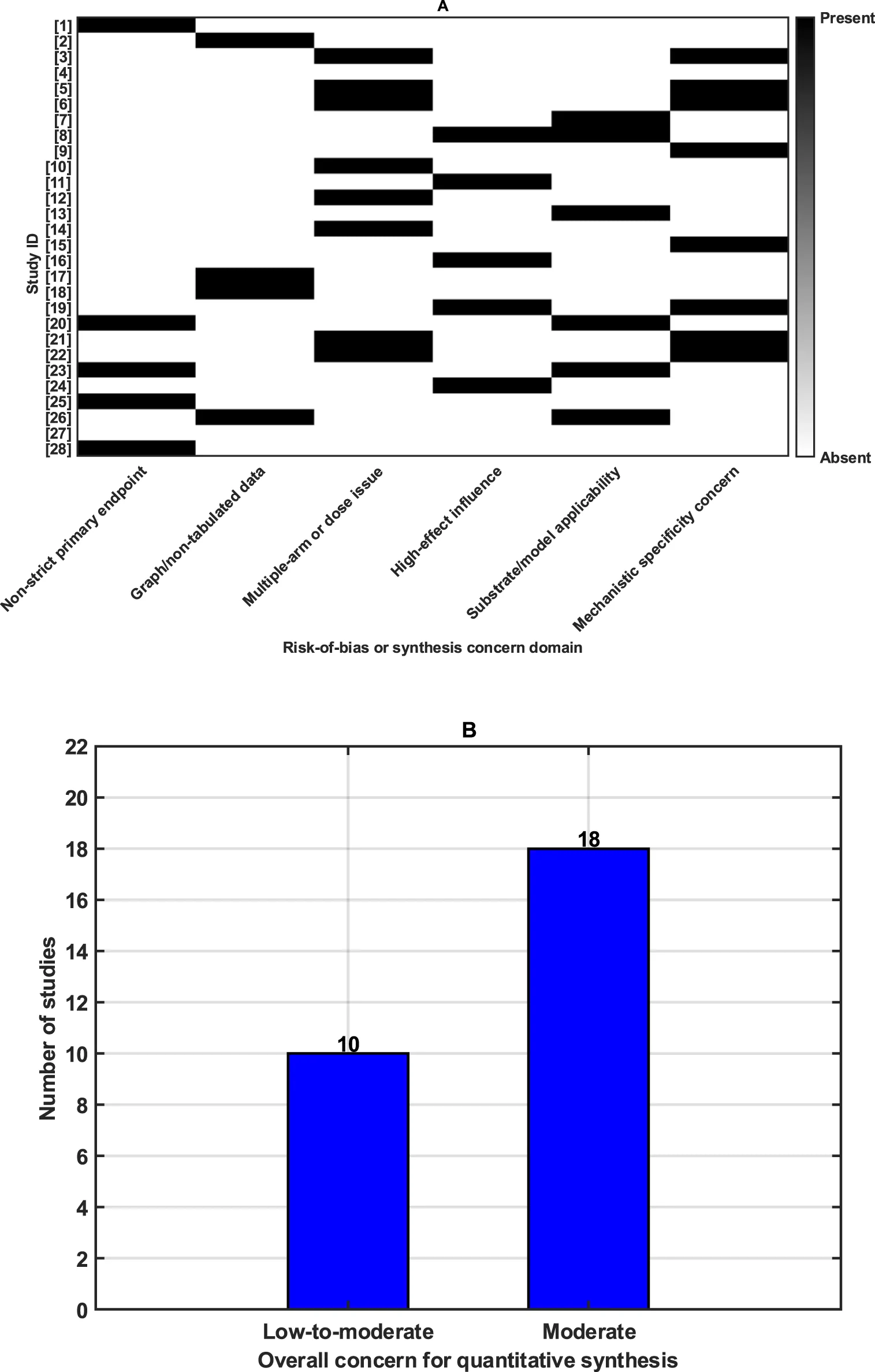
Study-level risk-of-bias and synthesis-concern profile. **(A)** Methodological and synthesis concern domains across the 28 included studies. **(B)** Distribution of overall concern levels for quantitative synthesis. In panel A, row numbers are sequential visualization study IDs rather than bibliography citation numbers.

The frequency summary in Figure 8B further clarifies the overall quality profile of the included studies. Most studies fell into the moderate concern category, whereas a smaller proportion were judged as low-to-moderate concern for quantitative synthesis. This distribution suggests that the evidence base was methodologically usable, but not free of complexity. Importantly, the predominance of moderate concern should not be interpreted as indicating invalid or unreliable findings; rather, it reflects the inherent challenges of synthesizing *in vitro* MDR-reversal studies that differ in resistant subline, metabolite class, concentration range, endpoint definition, and mechanistic depth.

Table 16 provides a more detailed explanation of the major methodological concerns affecting synthesis. Five studies lacked a directly comparable ADM/DOX IC50- or RF-based primary endpoint and were therefore excluded from the strict quantitative pool, although they remained informative for mechanistic interpretation. Four studies required graph-based extraction or did not present reversal outcomes in a directly tabulated format, which limited their suitability for primary pooling and justified a more cautious approach to sensitivity assessment. High-effect estimates, defined here as preferred RF or reversal-index values of at least 10, were identified in five studies and were considered particularly important for influence analysis because of their potential to inflate the overall RF-based estimate. Other issues, such as potential metabolite or dataset overlap, non-anthracycline primary substrates, limited mechanistic validation, and subline/model complexity, were less frequent but still analytically relevant.

From an interpretive perspective, the quality assessment supports two main conclusions. First, the major limitations of the included studies were primarily related to comparability and feasibility of synthesis, rather than to a complete absence of relevant biological evidence. Second, the identified concerns were sufficiently transparent and structurally manageable to allow a valid RF-based quantitative synthesis, provided that sensitivity analyses and careful study-selection rules were applied. In this regard, the findings in Figure 8 and Table 16 are consistent with the analytical strategy adopted in this review, namely the use of a strict primary RF dataset combined with mechanistic narrative synthesis for less directly comparable studies.

### 3.8 Ethnopharmacological linkage between traditional sources, bioactive metabolites, and MDR-Reversal evidence

Table 17 maps the included MDR-reversal evidence onto an ethnopharmacological source–metabolite–mechanism framework. This mapping identified 15 traditionally used natural sources that were directly linked to the reviewed K562-resistant cell evidence, including medicinal plants, a medicinal fungus, and other natural sources with documented traditional or ethnomedicinal relevance. The highest quantitative MDR-reversal signals were concentrated in five source–metabolite pairs: *Ailanthus altissima*–ailanthone, *Bouea macrophylla*–pentagalloyl glucose, *Scutellaria baicalensis*–oroxylin A, *Dioscorea* spp.–dioscin, and *Pueraria lobata*–puerarin. These interventions showed RF values of 26.40, 23.70, 17.99, 17.67, and 12.81, respectively, indicating that the strongest chemosensitizing effects were traceable to metabolites derived from traditionally relevant medicinal sources. The quantitative MDR-signal values in Table 17 are descriptive preferred study-level estimates rather than the variance-weighted inputs used in Table 12. Where removal effects are shown, the baseline geometric mean RF of 6.19 is descriptive and should not be conflated with the inverse-variance pooled RF of 6.31.

**TABLE 17:** Ethnopharmacological linkage between traditionally used natural sources, active metabolites, and MDR-reversal mechanisms in resistant K562 models.

Validated traditional source (authority and family)	Traditional-use context relevant to ethnopharmacology	Metabolite/intervention in this review	Included K562 evidence	Quantitative MDR signal	Main mechanistic linkage	References
*Ailanthus altissima* (Mill.) Swingle [Simaroubaceae]	Traditionally used bark source in Asian medicine, including China, Korea, and India, for inflammatory and chronic conditions such as asthma, bleeding-related conditions, and other disorders.	Ailanthone	K562/A02; 2.0 µM; DOX reversal assay	RF = 26.40; highest preferred estimate; removal reduced geometric mean RF from 6.19 to 5.71	P-gp functional inhibition, reduced Rh123 efflux, increased DOX retention, and PI3K/Akt-linked MDR1/P-gp suppression	(Li et al., 2021; Wang et al., 2014b)
*Bouea macrophylla* Griff. [Anacardiaceae]	Southeast Asian edible and medicinal plant with documented local/traditional use and reported antioxidant, anticancer, antihyperglycemic, and antimicrobial activities.	Pentagalloyl glucose	K562/ADR; 50 μg/mL; DOX reversal and P-gp efflux assays	RF = 23.70; high-effect estimate; removal reduced geometric mean RF from 6.19 to 5.74	P-gp transcript/protein suppression, inhibition of P-gp-mediated THP efflux, increased intracellular drug accumulation	(Li et al., 2019; Fu’adah et al., 2022)
*Scutellaria baicalensis* Georgi [Lamiaceae]	Traditional Chinese medicinal botanical drug historically used for inflammation, infection-related disorders, and other conditions; modern reviews report anticancer and anti-inflammatory pharmacology.	Oroxylin A; wogonin	Oroxylin A: K562/ADM, 15–60 μM; wogonin: K562/A02, 10–40 µM	Oroxylin A RF = 17.99; wogonin RF = 3.85	Oroxylin A: CXCL12/CXCR4 and PI3K/Akt/NF-κB suppression; wogonin: MRP1 downregulation through Nrf2/ARE inhibition	(Wang et al., 2022; Wang et al., 2014a; Zhao et al., 2019; Chanchal et al., 2023)
*Dioscorea* spp. [Dioscoreaceae; species-level source not reported]	Traditionally used *Dioscorea* species are medicinal and food plants with reported ethnomedicinal use in inflammatory, metabolic, and gastrointestinal disorders.	Dioscin	K562/ADR; 0.3 µM; ADR reversal assay	RF = 17.67; high-effect estimate; removal reduced geometric mean RF from 6.19 to 5.84	NF-κB-dependent MDR1/P-gp downregulation, inhibition of IκB-α phosphorylation/degradation, increased ADR accumulation	(Cai et al., 2019; Wang et al., 2024b)
*Pueraria montana* var. *lobata* (Willd.) Maesen and S.M.Almeida ex Sanjappa & Predeep [Fabaceae]	Traditional Chinese medicinal and edible root used in long-standing materia medica practice; commonly discussed for fever, metabolic, cardiovascular, and inflammatory indications.	Puerarin	K562/ADR; 25 μM; ADR sensitization and accumulation assays	RF = 12.81; high-effect estimate; removal reduced geometric mean RF from 6.19 to 5.94	NF-κB/IκB-α inhibition, MDR1/P-gp suppression, increased ADR accumulation, autophagy induction	(Han et al., 2018; Kou et al., 2025)
*Euphorbia lathyris* L. [Euphorbiaceae]	Traditional Chinese medicinal seed source; documented traditional use includes hydropsy, ascites, amenorrhea, constipation, scabies, schistosomiasis, and snakebite, with toxicity requiring caution.	Euphorbia factor L1	K562/ADR; 2.5–10.0 µM; DOX/VCR reversal assays	RF = 5.88; preferred RF used in strict synthesis	Functional ABCB1/P-gp inhibition, increased DOX/Rh123 accumulation, reduced Rh123 efflux, apoptosis sensitization without ABCB1 protein downregulation	(Li et al., 2012; Zhang et al., 2017; Wang et al., 2025b)
*Cnidium monnieri* (L.) Cusson [Apiaceae]	Widely used traditional botanical drug in China, Vietnam, and Japan; fruits used for diverse traditional indications.	Osthole	K562/ADM; 5 and 15 μM; DOX reversal and Rh123 assays	RF = 3.84 at 15 µM	P-gp/MDR1 downregulation, increased Rh123 and DOX accumulation, PI3K/Akt pathway inhibition	(Zhang et al., 2013; Li YiMin et al., 2015)
*Rheum palmatum* L. [Polygonaceae]	Rhubarb is an important traditional medicinal source; modern reviews discuss traditional use and pharmacology, including anthraquinone-rich metabolites.	Emodin	K562/ADM; 20 µM selected for conservative extraction	RF = 4.91; 40 µM RF = 9.30 reported but not selected as the conservative primary estimate	Competitive P-gp inhibition, P-gp expression downregulation, increased Rh123 uptake, apoptosis enhancement	(Min et al., 2017; Miao et al., 2025)
*Solanum nigrum* L. [Solanaceae]	Traditional medicinal plant with reported use in Asian traditional medicine and documented antitumor, anti-inflammatory, and hepatoprotective pharmacological evidence.	Solanine; S-20	Solanine: K562/ADM, 5–10 μg/mL; S-20: K562/ADR anti-MDR model	Solanine RF = 3.71; S-20 was mechanistic/non-strict RF evidence	Solanine: MRP1 downregulation via JNK-associated regulation; S-20: autophagy/ERK-associated reduction of BCRP, P-gp, and MRP1	(Xu et al., 2014; Ma et al., 2017; Zhang et al., 2023)
*Anemarrhena asphodeloides* Bunge [Asparagaceae]	Famous Chinese materia medica used in traditional Chinese medicine for more than two thousand years; traditionally associated with febrile, thirst-related, and metabolic indications.	Timosaponin A-III	K562/ADM; 1–2 μM; ADM/Rho-123/CFDA accumulation assays	RF = 2.52	Dual MDR1/P-gp and MRP1 suppression, increased transporter-substrate accumulation, PI3K/Akt inhibition	(Ji et al., 2011; Lin et al., 2020)
*Curcuma comosa* Roxb. [Zingiberaceae]	Traditional Thai medicinal rhizome used for gynecological and menstrual-related symptoms; diarylheptanoids are characteristic bioactive metabolites.	DHH	K562/ADR; DHH + DOX; transporter kinetic assays	Approximate RF = 2.84	Functional P-gp modulation, reduced THP efflux, increased intracellular drug accumulation, apoptosis enhancement without P-gp expression reduction	(Shen et al., 2025; Ganapathy et al., 2019; Su et al., 2012)
*Schisandra chinensis* (Turcz.) Baill. [Schisandraceae]	Traditional Chinese medicinal fruit, “Wu Wei Zi,” used as a tonic and for respiratory, hepatic, and vitality-related indications; lignans are major bioactive metabolites.	Schizandrol A	K562/A02; non-toxic range below 20 μM; DOX accumulation and Rh123 efflux assays	Graph-based/non-tabulated sensitization; not used as a strict primary RF estimate	P-gp functional inhibition, increased DOX accumulation, reduced Rh123 efflux, P-gp/p-Akt downregulation	(Li et al., 2019; Skalski et al., 2025)
*Ganoderma lucidum* (Curtis) P.Karst. [Ganodermataceae]	Traditional Chinese medicinal fungus, “Lingzhi/Reishi,” historically used for vitality, immune support, and chronic-disease contexts; triterpenoids are major metabolites.	Ethyl lucidenates A	K562/A02; 2.5–10 μM; VCR resistance model	Non-ADM/DOX endpoint; excluded from strict RF pool	P-gp functional inhibition, increased Rh123 accumulation, VCR resistance reversal without P-gp protein downregulation	(Yi et al., 2021; Hemdan and El-Shiekh, 2025; Wang et al., 2020)
*Tripterygium wilfordii* Hook.f. [Celastraceae]	Potent traditional Chinese medicinal source used historically for inflammatory and autoimmune disorders; toxicity is clinically relevant.	Triptolide	K562/A02; 5 nmol/L; ADM sensitization and MDR1 promoter assays	Graph-based/non-tabulated quantitative sensitization; retained as mechanistic evidence	MDR1/P-gp transcriptional suppression, reduced MDR1 promoter activity, increased intracellular ADM accumulation	(Li et al., 2010; Shan et al., 2023)
*Tanacetum parthenium* (L.) Sch.Bip. [Asteraceae]	Feverfew is widely used in traditional medicine for fever, migraine, inflammatory pain, and related conditions.	Parthenolide	K562/ADM and K562/ADM-derived resistant leukemia stem-like cells; DOX co-treatment	Graph-based/non-strict RF evidence; retained for mechanistic synthesis	NF-κB/P-gp suppression, ROS and mitochondrial-dependent apoptosis, targeting of resistant leukemia stem-like subpopulations	(Chen et al., 2016; Liu et al., 2023; Deng and Latte-Naor, 2018)

The moderate-effect evidence further supported this ethnopharmacological linkage. *Euphorbia lathyris*–derived Euphorbia factor L1 showed a preferred RF of 5.88. In contrast, emodin from *Rheum palmatum*, wogonin from *S. baicalensis*, osthole from *Cnidium monnieri*, solanine from *Solanum nigrum*, DHH from *Curcuma comosa*, and timosaponin A-III from *Anemarrhena asphodeloides* showed RF values or approximate reversal estimates ranging from 2.52 to 4.91. Several additional traditionally relevant sources, including *Schisandra chinensis*, *Ganoderma lucidum*, *Tripterygium wilfordii*, and *Tanacetum parthenium*, contributed mainly mechanistic rather than strict ADM/DOX RF evidence.

The source metabolite pairs converged on established MDR-reversal pathways. The strongest and moderate-effect interventions were primarily associated with P-gp/MDR1 inhibition or downregulation, increased intracellular anthracycline accumulation, reduced Rh123 or THP efflux, MRP1 or BCRP modulation, and suppression of NF-κB/IκB-α, PI3K/Akt, Nrf2/ARE, JNK, ERK, autophagy-related, or apoptosis-related resistance pathways. Therefore, Table 17 provides a direct response to the ethnopharmacological scope of the review by linking traditional medicinal sources to their bioactive metabolites and to experimentally validated mechanisms of MDR reversal in adriamycin- or doxorubicin-resistant K562 models.

## 4 Discussion

This systematic review and inverse-variance random-effects meta-analysis identified an overall IC50-based chemosensitization signal across a restricted set of adriamycin- or doxorubicin-resistant K562 experiments, while pharmacological interpretation remains specific to each metabolite, dose, resistant subline, substrate drug, and molecular target. In the primary meta-analysis of 19 study-level IC50-based effects, the pooled RF was 6.31 (95% CI, 4.77–8.36), summarizing the common IC50-based chemosensitization endpoint across the eligible comparisons. However, between-study heterogeneity was high, indicating that the magnitude of reversal varied substantially across resistant-cell sublines, metabolite classes, substrate drugs, and experimental conditions. The highest study-level estimates were observed for ailanthone, pentagalloyl glucose, oroxylin A, dioscin, and puerarin, whereas several other interventions produced moderate but still biologically relevant reversal effects (Wang Y. et al., 2014; Liu et al., 2021; Wang et al., 2013; Han et al., 2018; Dechsupa et al., 2023). These findings support further evaluation of the individual metabolites as MDR-modifying candidates, but they do not establish a uniform pharmacological action for natural products as a class.

Compared with earlier K562 MDR studies, the present synthesis extends the literature by placing metabolite-specific observations within a common IC50-based quantitative framework without treating chemically distinct metabolites as a single pharmacological class. Earlier studies in K562/A02 and related resistant sublines mainly showed that individual metabolites could reduce P-gp/MDR1 activity, increase intracellular ADR/DOX or Rh123 accumulation, or suppress MDR1/P-gp expression (Li et al., 2012; Ji et al., 2011; Shu-Qi et al., 2011). Similar mechanistic patterns were later reported in K562/ADM and K562/ADR models, where MDR reversal was associated not only with transporter inhibition but also with PI3K/Akt, NF-κB, JNK, Nrf2/ARE, apoptosis-related, and autophagy-related regulation (Chen et al., 2016; Xu et al., 2014; Min et al., 2017). The present meta-analysis is consistent with those earlier findings, but it adds a pooled IC50-based RF estimate for the common chemosensitization endpoint across eligible resistant K562 experiments; this pooled endpoint does not imply that the underlying mechanisms are shared across metabolites. This interpretation is also aligned with recent broader MDR literature showing that natural products can modulate ABC transporters by reducing protein expression, downregulating mRNA expression, inhibiting drug efflux, and affecting indirect regulatory pathways such as NF-κB, PI3K/Akt, ATPase-related activity, Nrf2, and apoptosis-related signaling (Sagadevan et al., 2026; Ma et al., 2026). However, unlike broader reviews that include multiple cancer types and mixed experimental models, the present analysis remains restricted to resistant K562 leukemia models and directly comparable ADM/ADR/DOX IC50-based reversal outcomes. Therefore, the current findings support the earlier mechanistic literature while providing a more focused quantitative estimate for K562-based MDR reversal.

Quantitatively, the pooled reversal fold of 6.31 lies within the moderate-to-high range of the earlier metabolite-specific reports, whereas the 95% prediction interval of 1.66–23.96 captures their marked dispersion. Thus, the present synthesis confirms the direction of the earlier mechanistic findings but tempers interpretation of the largest single-metabolite effects by showing that those estimates are not representative of the full K562 evidence base.

Accordingly, the pooled RF should not be interpreted as a metabolite-specific potency estimate for any single metabolite or botanical source. Rather, it provides a quantitative summary of the direction and approximate magnitude of IC50-based chemosensitization across a predefined, comparable subset of *in vitro* K562 MDR studies, while mechanistic and pharmacological differences are addressed through subgroup, sensitivity, and narrative synthesis.

The ethnopharmacological mapping presented in Table 17 strengthens the interpretation of the present findings by linking the experimental MDR-reversal evidence to traditionally used medicinal sources rather than treating the included interventions as isolated natural product-derived metabolites. Several high-effect metabolites, including ailanthone, pentagalloyl glucose, oroxylin A, dioscin, and puerarin, were derived from sources with documented traditional or ethnomedicinal relevance, such as *Ailanthus altissima*, *Bouea macrophylla*, *Scutellaria baicalensis*, *Dioscorea* spp., and *Pueraria lobata* (Li et al., 2021; Wang Y. et al., 2024; Kou et al., 2025; Zhang et al., 2017; Wang et al., 2025b). Similarly, moderate-effect or mechanistically important agents from *Euphorbia lathyris*, *Cnidium monnieri*, *Solanum nigrum*, *Curcuma comosa*, *Schisandra chinensis*, *Ganoderma lucidum*, *Tripterygium wilfordii*, and *Tanacetum parthenium* further support a source-based pharmacological rationale for MDR modulation (Li YiMin et al., 2015; Ganapathy et al., 2019; Deng and Latte-Naor, 2018). This linkage does not suggest that these traditional medicines were historically used to treat multidrug resistance or K562 leukemia specifically; rather, it indicates that traditionally relevant natural sources may contain bioactive metabolites capable of modulating modern resistance phenotypes through ABC-transporter regulation, improved intracellular anthracycline retention, apoptosis restoration, autophagy regulation, and suppression of NF-κB, PI3K/Akt, and Nrf2/ARE signaling pathways.

The quantitative findings should be interpreted in light of the model-specific distribution of effects. In the descriptive RF distribution, K562/A02 contributed the most study-level effects and showed the broadest response range, extending from modest reversal estimates to the high-effect estimate observed for ailanthone (Wang B. et al., 2016; Han et al., 2018). In the random-effects subgroup analysis, K562/A02 showed a pooled RF of 5.71, whereas K562/ADM showed 4.90. Within K562/ADM studies, oroxylin A represented the strongest study-level estimate in this subgroup (Chen et al., 2016; Wang Y. et al., 2014; Min et al., 2017; Wang H. et al., 2016; Yi et al., 2018). K562/ADR showed the highest subgroup-level pooled RF of 8.91, largely because several studies in this model reported high-effect interventions, including dioscin, puerarin, and pentagalloyl glucose (Liu et al., 2021; Wang et al., 2013; Dechsupa et al., 2023). This pattern suggests that the apparent magnitude of MDR reversal is influenced not only by metabolite identity but also by resistant-cell phenotype, transporter dependence, substrate drug, and dose-selection strategy.

Mechanistically, transporter modulation was the dominant explanatory theme. P-gp/MDR1 downregulation or functional inhibition was reported across a large subset of studies, including triptolide, archangelicin, timosaponin A-III, dioscin, osthole, resveratrol, emodin, ailanthone, schizandrol A, costunolide, puerarin, pentagalloyl glucose, parthenolide, and TMI (Li et al., 2012; Chen et al., 2016; Shu-Qi et al., 2011; Yi et al., 2021; Shen et al., 2025; Min et al., 2017; Wang L. et al., 2016; Han et al., 2018; Dechsupa et al., 2023). Importantly, the evidence suggests at least two transporter-centered phenotypes. Some agents reduced MDR1/P-gp expression or transcriptional activity, whereas others primarily inhibited transporter function without reducing P-gp abundance. Riccardin F, pakyonol, Euphorbia factor L1, ethyl lucidenates A, and DHH support the latter pattern, showing increased substrate accumulation or reduced efflux with limited evidence of P-gp expression suppression (Zhang et al., 2013; Ji et al., 2011; Li et al., 2019; Viriyaadhammaa et al., 2022). This distinction is important because direct transporter blockade and transcriptional transporter suppression may have different pharmacological implications.

The review also indicates that MDR reversal extends beyond P-gp. MRP1/ABCC1 modulation was implicated in wogonin, timosaponin A-III, solanine, and S-20 studies, while BCRP/ABCG2-related resistance was relevant in S-20, parthenolide, and TMI studies (Chen et al., 2016; Xu et al., 2014; Wang et al., 2022; Yi et al., 2021; Shen et al., 2025; Yi et al., 2018). These findings are consistent with a multidimensional resistance phenotype in which resistant K562 cells may rely on several ABC transporters and stress-response pathways rather than a single efflux mechanism. The Nrf2/ARE axis was particularly relevant for wogonin and TMI, linking antioxidant defense signaling with transporter regulation and resistance reversal (Xu et al., 2014; Shen et al., 2025). Similarly, solanine suggested an MRP1-centered mechanism associated with JNK signaling (Yi et al., 2018).

Cell death restoration and survival pathway inhibition represented another major interpretive theme. Several interventions increased apoptosis or restored drug-induced apoptotic responsiveness, including Haishengsu, triptolide, Euphorbia factor L1, oroxylin A, wogonin, timosaponin A-III, emodin, costunolide, parthenolide, DHH, and TMI (Li et al., 2012; Zhang et al., 2013; Chen et al., 2016; Xu et al., 2014; Yi et al., 2021; Shen et al., 2025; Li et al., 2010; Wang Y. et al., 2014; Min et al., 2017; Cai et al., 2019). PI3K/Akt-related signaling was the most recurrent pathway category, appearing in studies of oroxylin A, wogonin, timosaponin A-III, osthole, ailanthone, schizandrol A, costunolide, and resveratrol-bestatin (Chen et al., 2016; Wang Y. et al., 2014; Cai et al., 2019; Wang L. et al., 2016; Wang H. et al., 2016; Han et al., 2018). NF-κB/IκB-α signaling was also important, particularly where MDR1/P-gp transcriptional suppression coincided with enhanced chemosensitivity or apoptosis, as shown for oroxylin A, dioscin, puerarin, and parthenolide (Yi et al., 2021; Wang Y. et al., 2014; Liu et al., 2021; Wang et al., 2013). These findings support a model in which natural products reverse MDR through coordinated effects on efflux transporters, intracellular drug retention, survival signaling, and apoptotic execution.

Several studies broadened the mechanistic landscape beyond classical transporter reversal. Puerarin and S-20 linked MDR reversal to autophagy-associated processes, with S-20 providing evidence for cytotoxic autophagy and an ERK-related reduction in resistance protein levels (Wang et al., 2022; Liu et al., 2021). Parthenolide extended the discussion to drug-resistant leukemia stem-like populations by suppressing NF-κB/P-gp and enhancing DOX-induced apoptosis in the K562/ADM-derived resistant context (Yi et al., 2021). TMI provided a newer Nrf2-centered mechanism involving downregulation of P-gp and BCRP together with G2/M arrest and caspase-dependent apoptosis (Shen et al., 2025). These studies suggest that future MDR-reversal strategies may need to integrate transporter inhibition with targeting adaptive stress signaling, stem-like resistance phenotypes, and programmed cell death pathways.

Recent evidence further strengthens the contextual relevance of the present K562-focused synthesis. A recent review of ABC transporter-mediated resistance showed that natural products can modulate P-gp, BCRP, and MRP1 through suppression of protein expression, downregulation of mRNA expression, inhibition of drug efflux, and indirect regulation of NF-κB, PI3K/Akt, ATPase-related activity, and other resistance-associated pathways (Sagadevan et al., 2026). A complementary 2026 review focusing on terpenoids emphasized that monoterpenes, sesquiterpenes, diterpenes, and triterpenes may reverse ABC transporter-mediated MDR by blocking efflux pumps and regulating PI3K-AKT, Nrf2, and NF-κB signaling (Ma et al., 2026). Importantly, a recent leukemia-based case example using doxorubicin-resistant HL-60/DOXO cells showed that selected natural flavonoids, particularly apigenin, chrysin, and luteolin, could overcome doxorubicin resistance in association with Nrf2-related regulation, apoptosis, DNA damage, and ROS responses (Wadowski and Woźniak, 2025). Although this leukemia model is not K562 and was therefore not eligible for the primary quantitative pool, it supports the broader mechanistic relevance of Nrf2-linked chemosensitization and apoptosis restoration observed in the present synthesis. Recent broader reviews also support the role of natural products as multi-target MDR modulators, including metabolites such as curcumin, resveratrol, baicalein, chrysin, and plant-derived P-gp modulators from sesquiterpenoid, diterpenoid, chalcone, riccardin, and diarylheptanoid classes (Chen et al., 2024; Dinić et al., 2024). These recent case examples and reviews strengthen the translational rationale of natural product-derived MDR reversal while confirming that the pooled RF estimate should remain restricted to directly comparable IC50-based ADM/ADR/DOX reversal outcomes in resistant K562 sublines.

Four representative case studies illustrate the biological meaning of the pooled signal. Costunolide combined with doxorubicin increased apoptosis to 37.56% compared with 9.09% for doxorubicin alone while suppressing PI3K/Akt/mTOR signaling (Cai et al., 2019). Dioscin produced a 15.14-fold reversal in K562/ADR cells through NF-κB-dependent MDR1 suppression and recovery of intracellular adriamycin (Wang et al., 2013). In K562/A02 cells, ailanthone produced a 21.14-fold reversal together with P-gp efflux inhibition and PI3K/Akt-linked MDR1 downregulation (Han et al., 2018). Pentagalloyl glucose produced a 20.84-fold reversal in K562/ADR cells by jointly reducing P-gp expression and transport activity (Dechsupa et al., 2023). Taken together, these cases show three convergent strategies—functional efflux blockade, transcriptional transporter suppression, and restoration of drug-induced apoptosis—and identify testable candidates for validation in primary or patient-derived leukemia models.

The sensitivity analyses clarify the robustness and limitations of the pooled quantitative signal. Excluding high-effect studies from (Wang Y. et al., 2014; Liu et al., 2021; Wang et al., 2013; Han et al., 2018) and (Dechsupa et al., 2023) reduced the pooled RF from 6.31 to 4.51, but the reversal effect remained positive. Other sensitivity scenarios produced pooled RF values ranging from 6.09 to 6.97, indicating that the direction of the effect was stable across alternative assumptions. Leave-one-out analysis showed that omitting individual studies yielded pooled RF estimates ranging from 5.90 to 6.62. Omitting ailanthone (Han et al., 2018) and pentagalloyl glucose (Dechsupa et al., 2023) produced the largest downward shifts, whereas omission of lower-effect studies slightly increased the pooled estimate. These results indicate that no single study determined the overall conclusion, although high-effect studies meaningfully shaped the magnitude of the pooled estimate.

Small-study effect assessment also supported a cautious interpretation of the pooled estimate. The funnel plot showed mild right-sided asymmetry, but Egger’s test did not reach statistical significance, with p = 0.080. Trim-and-fill analysis imputed three studies and reduced the adjusted pooled RF to 5.48, with a 95% CI of 3.87–7.76. Therefore, the pooled chemosensitization signal remained positive after adjustment, although the possibility of small-study effects or selective reporting cannot be excluded in this preclinical literature.

The RF-based effect size also has important statistical limitations. RF is a ratio-based endpoint derived from IC50 values. It is therefore sensitive to differences in IC50 estimation methods, dose–response curve fitting, exposure duration, substrate drug, baseline resistance level, and the selected non-cytotoxic co-treatment concentration. Although log-transformation improves symmetry and allows interpretation of the pooled estimate as a geometric mean RF, it does not remove heterogeneity caused by different assay platforms or pharmacological designs. In addition, incomplete reporting of standard deviations, standard errors, replicate numbers, and confidence intervals limited the number of studies that could contribute to inverse-variance weighting and required exclusion or approximation of some otherwise relevant studies. RF also does not distinguish whether an apparent sensitization effect is driven by true MDR modulation, direct cytotoxicity of the tested intervention, reduced baseline IC50, or interaction with a specific substrate drug. Therefore, the pooled RF should be interpreted as an exploratory summary of relative chemosensitization under heterogeneous *in vitro* conditions, not as a standardized potency ranking or a directly translatable pharmacological effect size.

The main limitations arise from methodological heterogeneity, small experimental sample sizes, and incomplete standardization of outcome reporting across the included *in vitro* studies. First, although the primary IC50-based endpoint was analyzed using an inverse-variance random-effects meta-analysis, not all eligible studies reported sufficient variance data for inclusion in the pooled analysis; therefore, studies without directly usable ADM/ADR/DOX IC50-based effect-size and variance information were retained only for structured descriptive or mechanistic synthesis (Li et al., 2012; Wang et al., 2011; Wang S.-Q. et al., 2016; Xu et al., 2014; Wang et al., 2022; Wang et al., 2025a; Wang W.-z. et al., 2024; Lin et al., 2020; Wang B. et al., 2016). Second, several studies used multiple metabolites, multiple dose arms, or shared comparators, requiring predefined primary-arm extraction or sensitivity handling (Zhang et al., 2013; Chen et al., 2016; Ji et al., 2011; Wang et al., 2011; Wang S.-Q. et al., 2016; Xu et al., 2014; Wang Y. et al., 2014; Wang H. et al., 2016; Wang B. et al., 2016; Ma et al., 2017; Wang S.-Q. et al., 2014). Third, not all studies used ADM, ADR, or DOX as the primary substrate; VCR- and bestatin-based studies were mechanistically informative but were not appropriate for the primary anthracycline-based RF meta-analysis (Wang L. et al., 2016; Li et al., 2019). Fourth, some studies reported graph-based, screening-only, non-tabulated, or mechanistic outcomes rather than conventional IC50-based reversal, limiting direct comparability with the primary RF endpoint (Li et al., 2012; Wang et al., 2022; Yi et al., 2021; Shen et al., 2025; Li et al., 2010; Cai et al., 2019; Arken, 2019). These factors justify the combined use of a primary random-effects meta-analysis and structured mechanistic interpretation. Future research should prioritize complete IC50 data with standard deviations or standard errors, standardized resistant-cell validation, explicit definition of non-cytotoxic reversal concentrations, validated taxonomic and material-source reporting, consistent positive and negative controls, and confirmation of high-effect metabolites such as oroxylin A, puerarin, dioscin, ailanthone, and pentagalloyl glucose in additional leukemia models, primary resistant leukemia cells, and animal studies before clinical translation is considered (Yi et al., 2021; Sheng et al., 2024; Liu et al., 2021; Miao et al., 2025; Yi et al., 2018).

Accordingly, future MDR-reversal studies should report complete IC50 curve parameters, variance estimates, replicate numbers, viability thresholds for non-cytotoxic concentrations, and both absolute and ratio-based sensitization outcomes to improve the statistical robustness of future meta-analyses. Because CML treatment is clinically centered on BCR-ABL1 tyrosine kinase inhibition, the present findings should be interpreted as preclinical evidence of MDR modulation in resistant K562-derived models rather than as direct clinical evidence for CML treatment; future studies should therefore evaluate promising MDR-reversing metabolites in TKI-resistant, primary, and patient-derived leukemia models. Moreover, the clinical landscape of cancer treatment continues to evolve with immunotherapies, which may present their own challenges, such as pembrolizumab-induced autoimmune hemolytic anemia (Liu et al., 2026), underscoring the need for safe and effective therapeutic strategies (Qin et al., 2025).

In relation to Frontiers’ four-pillar expectations, the main reporting limitations of the original evidence base involved incomplete pharmacological standardization, variable reporting of dose selection and non-cytotoxic concentrations, incomplete taxonomic or source-material detail in some studies, inconsistent variance reporting for RF estimation, and the exclusive reliance on *in vitro* K562-derived models. These limitations were addressed in the present review by adding pharmacological evidence profiling, taxonomic/source validation, evidence hierarchy, quality-assessment criteria, and cautious interpretation restricted to preclinical *in vitro* MDR-reversal evidence.

This synthesis identifies a common IC50-based chemosensitization signal across the restricted quantitative dataset, while the pharmacological effects and mechanisms remain metabolite-specific. The strongest support comes from studies that combine IC50-based sensitization with functional evidence of increased intracellular drug accumulation, reduced efflux, transporter downregulation, apoptosis restoration, or pathway suppression. The pooled random-effects estimate summarized the common chemosensitization endpoint, but the high heterogeneity indicates that effect magnitude should be interpreted cautiously and in relation to resistant-cell subline, substrate drug, metabolite identity, and dose-selection strategy. Collectively, the findings identify individual metabolites with stronger study-level signals that warrant priority validation, particularly where sensitization was accompanied by experimentally demonstrated transporter or signaling effects. Future studies should report complete IC50 data with standard deviations or standard errors, use standardized resistant-cell validation, define non-cytotoxic reversal concentrations, distinguish transporter function from transporter expression effects, and confirm whether high-effect metabolites retain activity in more clinically relevant leukemia models. Future research could also explore their integration with advanced targeted delivery systems, such as carrier-free nanoassemblies for acute myeloid leukemia (Yang et al., 2025).

## 5 Conclusion

In the 19 eligible IC50-based comparisons, the common chemosensitization endpoint yielded a pooled RF of 6.31. This statistical summary does not represent a shared pharmacological action or potency across chemically distinct metabolites. The high heterogeneity indicates that effect magnitude varies substantially with metabolite identity, resistant-cell subline, substrate drug, dose selection, and experimental context.

Across individual studies, reported mechanisms included ABC transporter modulation, increased intracellular drug accumulation, reduced efflux, restoration of apoptosis, and changes in survival or stress-response pathways, including PI3K/Akt, NF-κB, and Nrf2/ARE signaling. Individual metabolites showing both chemosensitization and experimentally supported transporter or pathway effects warrant priority validation; no shared mechanism is inferred for the intervention class. Future studies should report complete IC50 statistics with variance estimates, use standardized resistant-cell validation, clearly define non-cytotoxic reversal concentrations, and confirm whether high-effect natural product-derived metabolites retain activity in more clinically relevant leukemia models.

## Data Availability

The original contributions presented in the study are included in the article/supplementary material, further inquiries can be directed to the corresponding author.
